# Identification of Hepatic Fibrosis and Steatosis via A Point‐of‐Care Transient Elastography System With Integrated AI

**DOI:** 10.1111/liv.70634

**Published:** 2026-04-08

**Authors:** Zi‐Hao Huang, Chen‐Hui Ye, Chong‐Lin Wu, Wan‐Rui Li, Miao‐Qin Deng, Li‐You Lian, Chen‐Xiao Huang, Yi‐Xuan Wei, Ying‐Ying Cao, Xiao‐Na Shen, Yi‐Wei Lin, Sui‐Dan Chen, Wai‐Kay Seto, Yong‐Ping Zheng, Ming‐Hua Zheng

**Affiliations:** ^1^ Department of Biomedical Engineering The Hong Kong Polytechnic University Hong Kong China; ^2^ MAFLD Research Center, Department of Hepatology The First Affiliated Hospital of Wenzhou Medical University Wenzhou China; ^3^ Department of Pathology The First Affiliated Hospital of Wenzhou Medical University Wenzhou China; ^4^ Department of Medicine, School of Clinical Medicine The University of Hong Kong Hong Kong China; ^5^ State Key Laboratory of Liver Research The University of Hong Kong Hong Kong China; ^6^ Department of Medicine The University of Hong Kong‐Shenzhen Hospital Shenzhen China; ^7^ Research Institute for Smart Ageing The Hong Kong Polytechnic University Hong Kong China; ^8^ Key Laboratory of Diagnosis and Treatment for the Development of Chronic Liver Disease in Zhejiang Province Wenzhou China

**Keywords:** fatty liver, liver elastography, liver stiffness measurement, point‐of‐care ultrasound, transient elastography, ultrasound attenuation

## Abstract

**Background & Aims:**

Transient elastography (TE) is routinely undertaken for non‐invasive assessment of liver fibrosis and steatosis, but is limited by its bulky design, inadequate imaging guidance and conventional algorithmic framework. Thus, we report a real‐time B‐mode image–guided, artificial intelligence–assisted, point‐of‐care TE (AI‐POC‐TE) system, providing simultaneous liver stiffness measurement (LSM) and a novel multi‐domain attenuation parameter (MAP) for fat quantification. We aimed to determine the accuracy of LSM and MAP in diagnosing histology‐confirmed fibrosis and steatosis in patients with chronic liver disease. Exploratory analyses assessed the minimum number of measurements required.

**Methods:**

This prospective study included 138 patients who underwent liver biopsy and AI‐POC‐TE simultaneously, and diagnostic performance was evaluated by area under the receiver operating characteristic curve (AUROC). Another larger cohort of 1455 patients was examined to benchmark AI‐POC‐TE against conventional TE (Fibroscan).

**Results:**

LSM by AI‐POC‐TE identified patients with fibrosis with AUROCs of 0.79 for ≥F2, 0.79 for ≥F3, 0.97 for F4. Corresponding Youden's cut‐offs were 8.2, 9.1 and 14.4 kPa. MAP detected steatosis of ≥ S1, ≥ S2, S3 with AUROCs of 0.92, 0.70, 0.76 and Youden's cut‐offs were 244, 278 and 294 dB/m, respectively. Among 1455 patients using both TE techniques, liver stiffness was highly correlated (*r* = 0.86) and MAP also correlated well with CAP (*r* = 0.80). Fewer than 10 measurements suffice to maintain accuracy; four measurements were statistically non‐inferior to the standard 10, supporting a streamlined protocol.

**Conclusion:**

We found AI‐POC‐TE to accurately assess fibrosis and steatosis, comparable to conventional TE but with added values of portability, B‐mode guidance and deep learning‐based analytics.

## Key Points

- We evaluated a newly developed, B‐mode image–guided, artificial intelligence–assisted, point‐of‐care transient elastography system (AI‐POC‐TE) for assessing liver fibrosis and steatosis.
- Using biopsy‐proven and paired TE cohorts, this system shows diagnostic performance comparable to conventional TE while providing advantages in size and AI‐based algorithmic analysis.
- Statistical non‐inferiority analyses demonstrate that accurate results can be achieved with as few as four measurements.

**Abbreviations**
AI: artificial intelligence
AI‐POC‐TE: artificial intelligence‐enabled point‐of‐care transient elastography
APRI: aspartate aminotransferase‐to‐platelet ratio index
ATI: attenuation imaging
AUROC: area under the receiver operating characteristic curve
BMI: body mass index
CAP: controlled attenuation parameter
CI: confidence interval
CLD: chronic liver disease
HSI: hepatic steatosis index
ICC: intraclass correlation coefficient
IQR: interquartile range
LSM: liver stiffness measurement
MAP: multi‐domain attenuation parameter
MASLD: metabolic dysfunction‐associated steatotic liver disease
NFS: NAFLD fibrosis score
POCUS: point‐of‐care ultrasound
RF: radiofrequency
ROC: receiver operating characteristics
ROI: region of interest
SCD: skin–liver capsule distance
TE: transient elastography
USG‐LSM: ultrasonography‐guided liver stiffness measurement

## 1 Background

Chronic liver disease (CLD) and cirrhosis account for approximately 2 million deaths worldwide each year [1]. Metabolic dysfunction‐associated steatotic liver disease (MASLD), affecting over 30% of adults, is now emerging as the predominant cause [2, 3]. Hepatic fibrosis and steatosis are the hallmarks of CLD across the disease spectrum, which necessitate accurate assessment. Biopsy remains the gold standard, but is invasive, costly and associated with morbidity [4]. Therefore, it is imperative to assess CLD in a manner that is non‐invasive and affordable by healthcare systems.

Transient elastography (TE), most commonly marketed as FibroScan (Echosens, Paris, France), is the best validated and widely used non‐invasive technology for CLD management [5, 6]. By means of vibroacoustic excitation, TE provides liver stiffness measurement (LSM) as a surrogate of liver fibrosis. Besides, liver fat can be simultaneously assessed by controlled attenuation parameter (CAP) [7], a quantitative imaging biomarker that measures the attenuation of ultrasonic signal through the liver. Major clinical practice guidelines from hepatology and endocrinology organizations recommended TE for routine patient care [8, 9, 10]. Nevertheless, technical challenges remain toward a more accessible and efficient examination procedure, as conventional TE possesses a bulky size, requires wired connection and lacks sufficient imaging guidance for liver localization [11, 12, 13, 14]. Furthermore, CAP relies on the attenuation analysis from a single ultrasonic beam rather than a larger 2D region of interest (ROI) [15, 16]. This limited sampling setting is considered a possible source of variability and inaccuracy in fat quantification. Existing TE also employed traditional algorithms that have remained noticeably less updated for decades. In this connection, another room to improve TE is the incorporation of artificial intelligence (AI) into data processing and analytics [17, 18, 19].

To mitigate the above concerns, a new TE framework has been developed with the integration of (1) AI, (2) wireless point‐of‐care ultrasound (POCUS) and (3) real‐time B‐mode imaging guidance. It is generally referred to as AI‐enabled, point‐of‐care TE (AI‐POC‐TE) in this study. AI‐POC‐TE allows simultaneous LSM and multi‐domain attenuation parameter (MAP, a refined algorithmic method compared to CAP) during B‐mode liver imaging to assess fibrosis and steatosis, respectively. To our best knowledge, the diagnostic accuracy of imaging‐guided LSM and ultrasound attenuation measurement has not yet been established in the literature, although conventional unguided methods are extensively studied [5, 6, 7, 8, 9]. Moreover, uncertainty remains about whether a downsized design would compromise diagnostic accuracy. Another related question is how many measurements suffice to ensure accurate TE, assuming the liver is “on‐target” while guided. The practice of acquiring 10 valid measurements is indeed established in the field [6]; however, it appears to be a common convention rather than a standard derived from systematic evaluation.

The primary objective of this study was to examine the diagnostic performance of AI‐POC‐TE, including LSM and MAP, for staging liver fibrosis and steatosis in patients with biopsy‐proven CLD. We further compared the performance of AI‐POC‐TE with other established non‐invasive tests, using histologic analysis as the reference standard. The secondary objective was to perform a prospective validation study between conventional TE and AI‐POC‐TE in a large cohort of over 1400 study participants. We hypothesized that AI‐POC‐TE is non‐inferior to conventional TE in terms of both liver fibrosis and fat quantification. Additionally, we sought to evaluate how the number of measurements affects accuracy, and to determine the minimum number and optimal combination of measurements required for LSM and MAP, respectively.

## 2 Methods

### 2.1 Introduction of AI‐Empowered, 2D Imaging‐Guided, POC‐TE System

**FIGURE 1:**
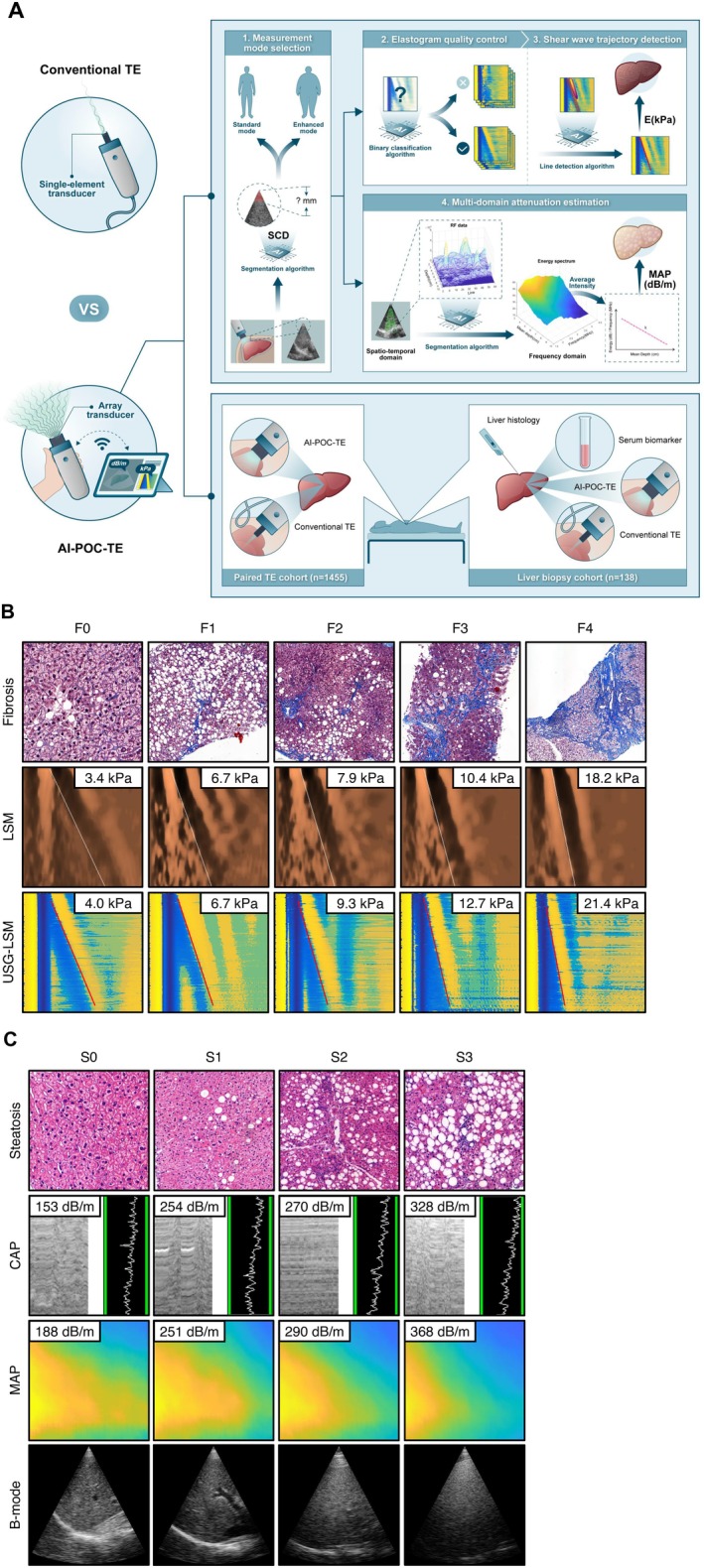
Study design overview. (A) Schematic representation of B‐mode ultrasound‐guided, AI‐enabled, point‐of‐care TE (AI‐POC‐TE): Both measures were collected simultaneously with B‐mode images, which is an explicit imaging‐guided measurement procedure of the liver; (B) Example results of AI‐POC‐TE in comparison of histology and conventional TE.

AI‐POC‐TE was conducted with a prototypical device developed under the Liverscan (Eieling Technology, Hong Kong, China) system framework [11, 12, 20]. An overview of the system and study design is provided in Figure 1.

#### 2.1.1 Hardware

The system comprises a TE probe and a mobile‐based terminal, which are wirelessly communicated via Wi‐Fi. The probe is made of a 50‐Hz vibrator, a 3.5‐MHz phased array transducer and miniaturized control electronics in a fully integrated format. The user interface displays real‐time B‐mode images, measurement results and control panels, while embedded deep learning algorithms process the acquired signals.

#### 2.1.2 Parameters and AI Algorithms

AI‐POC‐TE leverages the technological principles from both B‐mode ultrasonography and TE, where AI was applied to enhance its algorithmic framework. Parts of the work have been published [11, 12, 21]. This technique provided two investigational measures—ultrasonography‐guided LSM (USG‐LSM, in kPa) and MAP (in dB/m). Specifically, USG‐LSM utilized an elastogram to quantify shear wave propagation velocity at 50 Hz. Elastograms were constructed from temporal RF signals using a texture disturbance detection approach [11]. As part of quality control, a ConvNeXt V2–based deep learning model [22] was employed to assess elastogram quality. Criteria used to train this binary classifier (high‐ vs. poor‐quality elastograms) included signal intensity, length, linearity, continuity, parallelism of the primary wave trajectory, as well as background noise level. Subsequently, a Transformer‐enhanced Hough Transform–based deep learning model [21] was employed to identify the wave trajectory on high‐quality elastograms. Its slope was then used to estimate the Young's modulus of the liver.

Another deep learning algorithm was developed to autonomously determine an appropriate measurement mode for different patient morphotypes. Using the YOLOv8 model [23], subcutaneous tissues were segmented on B‐mode images to compute the midline skin‐liver capsule distance (SCD). Upon detecting an SCD > 20 mm, the software automatically activates the “enhanced” mode from the default “standard” mode to accommodate obese patients. Specific differences between modes include their B‐mode image depth (180 vs. 140 mm), overall gain (70 vs. 50 dB), peak‐to‐peak vibration amplitude (3 vs. 2 mm), and measurement range below the skin surface (35–75 vs. 25–65 mm).

MAP is an estimate of ultrasound attenuation over multiple sets of radiofrequency (RF) echo signals at 3.5 MHz, specifically targeting the liver parenchyma through a proprietary spatial averaging–spectral analysis approach. For this purpose, 256 RF lines were obtained from the 20–100 mm depth range to construct a spatial greyscale image. A BiSeNet V2–based deep learning model [24] was employed to segment the liver border, while a signal gradient–based denoising algorithm excluded heterogeneous signals, such as intra‐hepatic vessels. This combined approach allowed for the full extraction of liver parenchymal regions, which varied according to the probe's location or patients' morphotype. MAP quality control was conducted independently of LSM, the latter of which relied on elastogram characteristics; a mask qualified for subsequent frequency‐domain analyses if liver tissues constituted > 80% of the mask area. To eliminate operator or instrument setting dependencies, the power spectrum of different adjacent frequencies was normalized and finally calibrated against reference phantoms with known attenuation coefficients. MAP was computed from an average spectrum across the same depth range, with values ranging from 100 to 400 dB/m. Major improvements of MAP over CAP included an increased sampling volume (32 RF lines vs. single line), an optimized measurement depth (adaptive vs. fixed ROI), a comprehensive domain analysis (time–frequency vs. frequency domain), and stringent quality control (independent criteria vs. reliance on LSM quality).

### 2.2 Study Design and Participants

The single‐institution research generally contains two prospective study cohorts. The *biopsy* cohort was a diagnostic accuracy study. It was designed to identify the optimal USG‐LSM and MAP cut‐offs for evaluating fibrosis and steatosis, with the reference standard of liver biopsy. The *paired TE* cohort was another trial which adopted a head‐to‐head comparison design to validate AI‐POC‐TE against conventional TE; participants from this cohort underwent both TE examinations on the same day. Consecutive patients were recruited between October 2023 and September 2025 from a tertiary care liver clinic at The First Affiliated Hospital of Wenzhou Medical University. The hospital ethics committee approved this trial, and we followed the STARD guidelines to report study outcomes.

#### 2.2.1 Inclusion and Exclusion Criteria

We included adults with a spectrum of known or suspected chronic liver disease, who had (1) a scheduled liver biopsy or (2) a clinical indication for TE examination. Patients who underwent conventional TE and AI‐POC‐TE on the day of liver histology analysis were assigned to the *biopsy* cohort, whereas those with only concurrent TE data (without histologic data) comprised the *paired TE* cohort. Exclusion criteria for both cohorts were patients with ascites, active cardiac implant, prior liver transplantation, or refusal to take paired TE examinations. All eligible participants gave informed consent.

### 2.3 Histologic Examination

In the *biopsy* cohort, liver histology serves as the reference for determining the diagnostic accuracy of AI‐POC‐TE. Percutaneous liver biopsy procedure was performed using a 16‐gauge Hepafix needle. Specimens were fixed in formalin and stained with at least haematoxylin and eosin and Masson's trichrome. An experienced hepatopathologist independently analysed slides without knowledge of liver ultrasound and other clinical data. Biopsies shorter than 15 mm in length were deemed uninterpretable and excluded. For patients with MASLD, fibrosis (F0–F4) was staged according to the NASH Clinical Research Network scoring system. For patients with other liver disease contexts, including chronic hepatitis B (CHB), fibrosis was staged according to the Scheuer system. Steatosis and lobular inflammation were scored ordinally from 0 to 3, and hepatocellular ballooning was scored from 0 to 2.

### 2.4 Clinical Examination

All participants underwent a standardized clinical evaluation on the day of enrollment, including medical history, demographic and anthropometric examinations, and biochemical tests. Significant alcohol intake above the recommended limits (> 70 g/week for women and > 140 g/week for men) was documented. A fasting venous blood sample was taken for investigation of basic liver biochemistry and clinical laboratory parameters. In patients with complete biochemical data, the performance of LSM by AI‐POC‐TE was compared with the other three fully validated fibrosis biomarkers: the FIB‐4, the AST‐to‐platelet ratio index (APRI) and the NAFLD fibrosis score (NFS). Likewise, MAP was assessed against the hepatic steatosis index (HSI), a readily available composite score for detecting fatty liver.

### 2.5 TE Examination

#### 2.5.1 Conventional TE (FibroScan LSM and CAP)

Conventional TE was performed using the FibroScan system (Model Pro‐E) by an independent, experienced, certified technician blinded to the TE results of others. The new features such as SmartExam and continuous CAP were not available during the study period. All participants fasted for over 3 h before TE. Following the standard procedure described previously [5, 6], the transducer tip was placed onto an appropriate intercostal space over the right liver lobe, with patients lying in the dorsal decubitus position. The use of M or XL probe depends on an automatic probe selection tool embedded in the device. LSM and CAP by FibroScan were obtained simultaneously. Only examinations with ≥ 10 valid individual measurements were considered valid. The median of 10 valid LSM and CAP values were included for statistical analysis.

#### 2.5.2 AI‐POC‐TE (USG‐LSM and MAP)

In the session of AI‐POC‐TE using the prototypical AI‐POC‐TE system, the same examination procedure, including patient positioning and measurement protocol, was applied as outlined above. Five trained novice operators performed the AI‐POC‐TE examination as per the manufacturer's recommendations. The inter‐operator reliability of this technique has been previously established [11]. Guided by real‐time B‐mode, a sufficiently thick portion of the liver parenchyma free of large vessels was identified and consecutively measured 10 times. A single probe was designed for universal use across varying patient morphotypes. The standard or enhanced mode of measurement was automatically selected according to the real‐time SCD assessment, as indicated by the device software. Ten valid repetition measurements were recorded according to the outputs of the elastogram quality control algorithm. The median LSM and MAP were derived and used in this study as the per‐patient right‐lobe AI‐POC‐TE result.

### 2.6 Statistical Analysis

#### 2.6.1 Sample Size Estimation

The sample size was calculated for the primary objective of estimating the AI‐POC‐TE accuracy to achieve a clinically acceptable AUROC with a 5% standard error in the subgroup of biopsy study. Given an expected AUROC of 0.80 for detecting any steatosis (≥ 5% hepatocytes affected on histology) and an estimated 95% confidence interval (CI) of 0.74–0.86 (CI width: 0.125) at a prevalence of 30%, 107 participants were projected. The final sample size was set at 130 to allow for a 20% dropout.

#### 2.6.2 Descriptive Statistics

Cohort characteristics were descriptively summarized as median with interquartile range (IQR) for continuous variables and absolute figures with percentages for categorical variables. The Kruskal–Wallis test was used to compare liver stiffness and attenuation parameters between groups at different stages of fibrosis and grades of steatosis, respectively.

#### 2.6.3 Diagnostic Performance

Overall accuracy of AI‐POC‐TE‐based LSM and MAP was assessed using receiver operating characteristic (ROC) curve. We used Youden's index to estimate the optimal cut‐offs, together with its performance metrics: area under the receiver operating characteristic curve (AUROC), sensitivity, specificity, positive predictive value, and negative predictive value. Pearson's correlation coefficients were separately calculated for LSM versus fibrosis stage and MAP versus steatosis grade. Secondary analyses compared AI‐POC‐TE with conventional TE and serum biomarkers for identifying those clinically relevant events of fibrosis (≥ F2 and F4) and steatosis (≥ S1, ≥ S2 and S3). AUROC comparison between TE techniques was done using DeLong's test.

#### 2.6.4 Classification

Stage‐based concordance was evaluated using confusion matrices, overall agreement and weighted Cohen's kappa statistics.

#### 2.6.5 Validity Statistics

In the *paired TE* cohort, the association between the attenuation measure pair (CAP vs. MAP) was examined using Pearson's correlation and simple linear regression. Further analysis of agreement was performed by a Bland–Altman plot, with CAP as the benchmark technique. We also applied the same set of analyses to the LSM pair (LSM vs. USG‐LSM).

#### 2.6.6 Exploratory Research

To evaluate the effect of the number of measurements on accuracy, subgroup ROC analyses were conducted for USG‐LSM and MAP, respectively, using various median subsets (i.e., medians derived from 1 measurement to 10 measurements). AUROCs obtained using 1–9 measurements were compared with the reference AUROC derived from 10 measurements, and paired DeLong's tests were applied to evaluate statistical non‐inferiority. Additionally, intraclass correlation coefficient (ICCs), Spearman's rank correlation coefficient and mean difference were reported for each median subset in comparison with the reference subset of 10 measurements.

## 3 Results

In this prospective study, we screened 1603 potentially eligible participants, 146 of whom had undergone liver biopsy. The study flowchart is shown in Figure S1. Consequently, 138 and 1455 patients were included in the *biopsy* cohort and the *paired TE* cohort for analysis, respectively. Baseline characteristics for both populations are listed in Table 1, with overweight and obesity being more prevalent in the *biopsy* cohort. According to the automatic measurement mode selection algorithm, the enhanced mode of AI‐POC‐TE was applied to approximately 30% of biopsied patients. They had a significantly higher median BMI (30 vs. 26 kg/m^2^) and SCD (23 vs. 17 mm) compared to patients measured with the standard mode. Of these patients with paired conventional TE (FibroScan) results, 104 (75%) were evaluated with the M probe and 34 (25%) with the XL probe. The measurement failure rate was comparable between conventional TE and AI‐POC‐TE (18 of 1473 [1.2%] vs. 16 of 1473 [1.1%], *p =* 0.86) in the whole population.

**TABLE 1:** Baseline patient characteristics of the two study cohorts.

Variable	Biopsy cohort (*n* = 138)	Paired TE cohort (*n* = 1455)
Demographics		
Male	98 (71%)	977 (69%)
Age, years	47 [34–54]	46 [37–54]
Significant alcohol intake	22 (16%)	214 (15%)
Anthropometrics		
BMI, kg/m^2^	27.3 [24–29]	25.0 [23–28]
SCD, mm	18 [16–21]	17 [14–19]
Metabolic factor		
Overweight & obese	97 (70%)	754 (52%)
Diabetes	18 (13%)	164 (11%)
Hypertension	35 (25%)	317 (22%)
Liver disease aetiology		
Viral (CHB, CHC)	19 (14%)	622 (43%)
MASLD	110 (80%)	572 (39%)
ALD	5 (4%)	73 (5%)
Other	4 (3%)	188 (13%)
Imaging biomarker		
LSM by conventional TE, kPa	7.1 [5.8–9.5]	6.1 [5.0–7.8]
CAP by conventional TE, dB/m	281 [232–314]	258 [212–303]
XL probe of conventional TE	34 (25%)	263 (18%)
USG‐LSM by AI‐POC‐TE, kPa	8.5 [6.4–11.0]	6.8 [5.3–8.9]
MAP by AI‐POC‐TE, dB/m	302 [270–339]	286 [248–327]
Enhanced mode of AI‐POC‐TE	37 (27%)	210 (14%)
Blood		
Albumin, mg/dL	46 [43–48]	46 [44–48]
ALT, IU/L	52 [31–94]	36 [24–72]
AST, IU/L	39 [27–66]	30 [23–44]
Triglyceride, mmol/L	1.7 [1.1–2.6]	1.7 [1.1–2.5]
Fasting glucose, mmol/L	5.5 [4.9–6.3]	5.4 [4.9–6.1]
HbA1c, %	5.9 [5.5–6.2]	5.5 [5.5–6.3]
Platelet count, ×10^9^/L	230 [184–276]	225 [184–264]
FIB‐4	1.1 [0.7–1.8]	1.1 [0.7–1.5]
APRI	0.5 [0.3–0.8]	0.4 [0.3–0.6]
NFS	−2.6 [−3.5 to −1.3]	−1.9 [−3.1 to −0.4]
HSI	39 [35–44]	35 [32–40]
Fibrosis stage		
F0	15 (11%)	—
F1	57 (41%)	—
F2	39 (28%)	—
F3	17 (12%)	—
F4	10 (7%)	—
Steatosis grade		
S0	10 (7%)	—
S1	43 (32%)	—
S2	54 (40%)	—
S3	29 (21%)	—
Lobular inflammation grade		
I0	3 (2%)	—
I1	64 (48%)	—
I2	66 (49%)	—
I3	1 (1%)	—
Ballooning grade		
B0	16 (12%)	—
B1	61 (45%)	—
B2	58 (43%)	—

*Note:* All data are median [25th–75th percentile] or figure (percentage), unless otherwise indicated. The analyses include only patients with available data. Conventional TE was performed using the Fibroscan system.

Abbreviations: ALT, alanine aminotransferase; AST, aspartate aminotransferase; BMI, body mass index; Haemoglobin A1c, HbA1c; HDL, high‐density lipoprotein; LDL, low‐density lipoprotein; MASH, Metabolic dysfunction‐associated steatohepatitis; NAS, NAFLD Activity Score; SCD, skin‐liver capsule distance.

### 3.1 Accuracy of AI‐POC‐TE for Hepatic Fibrosis and Steatosis

**FIGURE 2:**
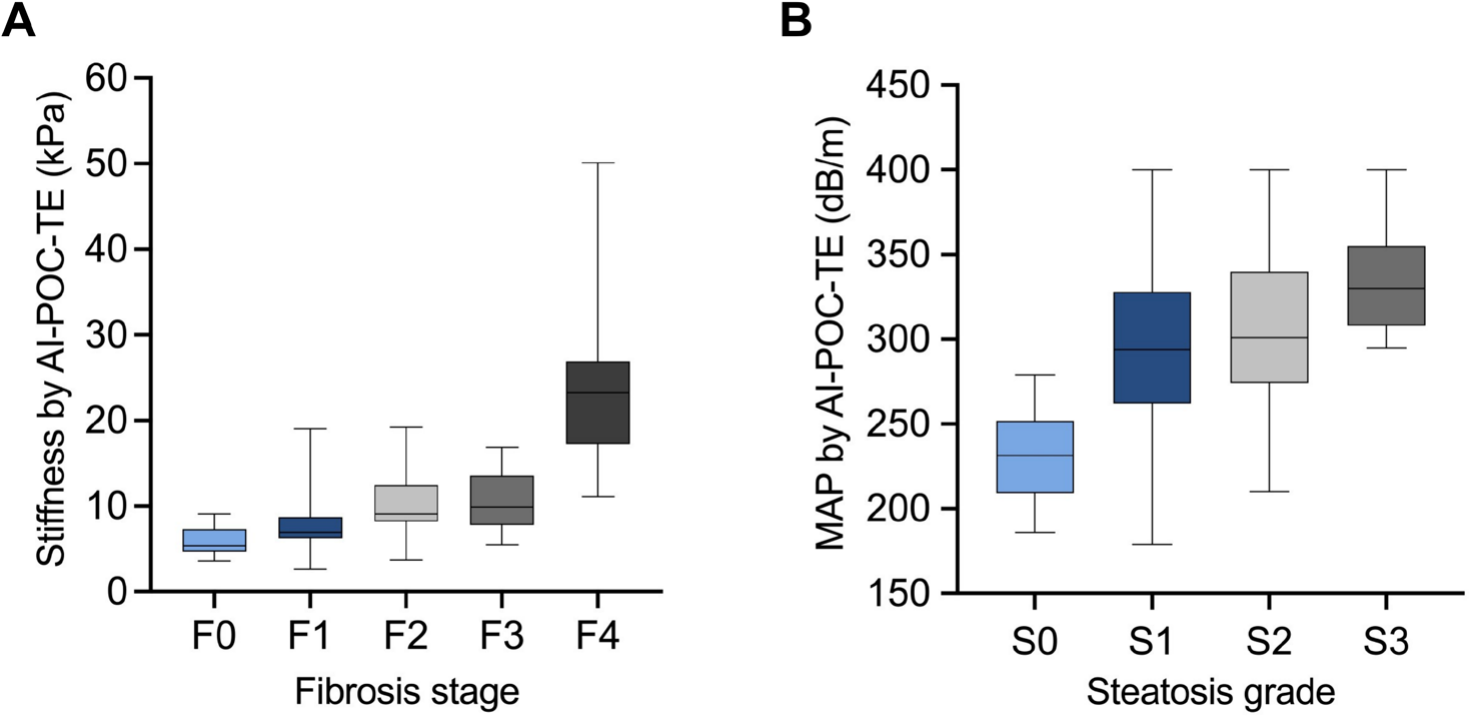
Distribution of (A) USG‐LSM values stratified by fibrosis stage (Kruskal‐Wallis test: *H* = 51.08, df = 4, *p* < 0.001; post hoc Dunn's test: *p* = 0.76 for F2 and F3, *p* < 0.05 otherwise); (B) MAP values stratified by steatosis grade (Kruskal–Wallis test: H = 34.25, df = 3, *p* < 0.001; post hoc Dunn's test: *p* = 0.26 for S1 and S2, *p* < 0.05 otherwise).

Of 138 patients with interpretable biopsies and valid USG‐LSM values, the fibrosis stage distribution was 11%, 41%, 28%, 12%, 7% for F0, F1, F2, F3, F4, respectively. The boxplot of USG‐LSM vs. fibrosis stage is shown in Figure 2A. USG‐LSM values showed a significant upward trend across fibrosis stages (Kruskal–Wallis *p* < 0.001). USG‐LSM values were moderately correlated with fibrosis stage (*r* = 0.60, *p* < 0.001), and had a weak correlation with ballooning grade (*r* = 0.23, *p* = 0.01). The AUROC of USG‐LSM in detecting ≥ F1, ≥ F2, ≥ F3 and F4 was 0.83 (95% CI: 0.75–0.89), 0.79 (95% CI: 0.71–0.86), 0.79 (95% CI: 0.72–0.86), and 0.97 (95% CI: 0.93–0.99), respectively. The corresponding optimal cut‐off values maximizing Youden's index were 5.8, 8.2, 9.1 and 14.4 kPa, respectively.

Of 136 patients with interpretable biopsies and valid MAP values, the steatosis grade distribution was 7%, 32%, 40%, 21% for S0, S1, S2, S3, respectively. MAP values significantly increased with increasing steatosis grade (Kruskal‐Wallis *p* < 0.001) with the exception of S1 and S2 (*p* = 0.26). There was a moderate positive correlation between MAP and steatosis grade (*r* = 0.47, *p* < 0.001). MAP also weakly correlated with ballooning grade (*r* = 0.21, *p* = 0.02) and inflammation grade (*r* = 0.28, *p* = 0.001). The AUROC of MAP in detecting steatosis ≥S1, ≥S2, and S3 was 0.92 (95% CI: 0.86–0.96), 0.70 (95% CI: 0.61–0.77) and 0.76 (95% CI: 0.68–0.83), respectively. Their corresponding optimal cut‐off values maximizing Youden's index were 244, 278 and 294 dB/m. The diagnostic statistics of AI‐POC‐TE at each optimal cut‐off are detailed in Table 2 and the ROC curve is given in Figure S2. We also provided the ROC results of conventional TE for the same cohort in Table S1.

**TABLE 2:** Diagnostic test characteristics of AI‐POC‐TE for hepatic fibrosis and steatosis.

	Prevalence	AUROC (95% CI)	Cut‐off selection criterion	Cut‐off, kPa or dB/m	Sensitivity	Specificity	PPV	NPV
USG‐LSM by AI‐POC‐TE	*n* = 138							
≥ F1	89%	0.83 (0.75–0.89)	Youden's index	5.8 kPa	0.89	0.67	0.96	0.43
			Fixed sensitivity ≥ 0.9	5.7 kPa	0.91	0.60	0.95	0.45
			Fixed specificity ≥ 0.9	8.8 kPa	0.46	0.93	0.98	0.18
≥ F2	48%	0.79 (0.71–0.86)	Youden's index	8.2 kPa	0.79	0.72	0.72	0.79
			Fixed sensitivity ≥ 0.9	6.0 kPa	0.91	0.31	0.55	0.79
			Fixed specificity ≥ 0.9	10.6 kPa	0.44	0.90	0.80	0.64
≥ F3	20%	0.79 (0.72–0.86)	Youden's index	9.1 kPa	0.74	0.72	0.40	0.92
			Fixed sensitivity ≥ 0.9	7.0 kPa	0.93	0.42	0.29	0.96
			Fixed specificity ≥ 0.9	12.6 kPa	0.52	0.90	0.57	0.88
F4	7%	0.97 (0.93–0.99)	Youden's index	14.4 kPa	0.90	0.93	0.49	0.99
			Fixed sensitivity ≥ 0.9	14.4 kPa	0.90	0.93	0.49	0.99
			Fixed specificity ≥ 0.9	13.7 kPa	0.90	0.91	0.43	0.99
MAP by AI‐POC‐TE	*n* = 136							
≥ S1	93%	0.92 (0.86–0.96)	Youden's index	244 dB/m	0.94	0.80	0.98	0.50
			Fixed sensitivity ≥ 0.9	255 dB/m	0.90	0.80	0.98	0.38
			Fixed specificity ≥ 0.9	276 dB/m	0.75	0.90	0.99	0.21
≥ S2	61%	0.70 (0.61–0.77)	Youden's index	278 dB/m	0.82	0.53	0.73	0.65
			Fixed sensitivity ≥ 0.9	264 dB/m	0.90	0.36	0.69	0.70
			Fixed specificity ≥ 0.9	349 dB/m	0.25	0.91	0.81	0.44
S3	21%	0.76 (0.68–0.83)	Youden's index	294 dB/m	1.00	0.54	0.37	1.00
			Fixed sensitivity ≥ 0.9	301 dB/m	0.93	0.60	0.38	0.97
			Fixed specificity ≥ 0.9	368 dB/m	0.17	0.91	0.33	0.80

Abbreviations: AI‐POC‐TE, artificial intelligence‐enabled point‐of‐care transient elastography; AUROC, area under the receiver operating characteristics curve; MAP, multi‐domain attenuation parameter; NPV, negative predictive value; PPV, positive predictive value; USG‐LSM, ultrasonography‐guided liver stiffness measurement.

To explore the impact of disease aetiology, subgroup analyses were performed in patients with MASLD and CHB (Table S2). The diagnostic performance and optimal cut‐offs of AI‐POC‐TE were broadly comparable across aetiologies.

### 3.2 Comparison of AI‐POC‐TE With Other Non‐Invasive Tests

Table 3 summarizes the inter‐technique comparisons of AUROC for four clinically significant histologic features: significant fibrosis (≥ F2), cirrhosis (F4), mild steatosis (≥ S1), moderate steatosis (≥ S2) and severe steatosis (S3). Direct comparisons showed that LSM by conventional TE was as accurate as AI‐POC‐TE for diagnosing cirrhosis, with no significant difference as assessed by DeLong's test (AUROC: 0.91 vs. 0.97, *p* = 0.40). Similar accuracy was observed for predicting ≥ F2; the two techniques showed comparable AUROCs (0.77 vs. 0.81, *p* = 0.18). Furthermore, the performance of AI‐POC‐TE was compared pairwise with other fibrosis biomarkers in 132 patients with available serologic data. USG‐LSM significantly outperformed FIB‐4 (AUROC = 0.65; *p* = 0.01) and NFS (AUROC = 0.60; *p* < 0.001) for the diagnosis of ≥ F2, and APRI (AUROC = 0.80; *p* = 0.01) for the diagnosis of F4.

**TABLE 3:** Comparative diagnostic accuracy of imaging versus serum biomarkers for clinically significant histologic findings.

	AUROC (95% CI)	Optimal cut‐off	Sensitivity	Specificity	PPV	NPV	*p*‐value^e^ (vs. AI‐POC‐TE), *p*
≥ F2	*n* = 132						
USG‐LSM by AI‐POC‐TE	0.81 (0.74–0.88)	8.5 kPa	0.76	0.80	0.78	0.78	—
LSM by conventional TE*	0.77 (0.69–0.84)	6.8 kPa	0.79	0.65	0.68	0.77	0.181
FIB‐4	0.65 (0.56–0.73)	1.03	0.65	0.61	0.61	0.65	0.009
APRI	0.73 (0.64–0.80)	0.67	0.51	0.87	0.78	0.66	0.079
NFS	0.60 (0.51–0.68)	−2.51	0.59	0.65	0.61	0.63	< 0.001
F4	*n* = 132						
USG‐LSM by AI‐POC‐TE	0.97 (0.92–0.99)	14.4 kPa	0.90	0.93	0.49	0.99	—
LSM by conventional TE*	0.91 (0.84–0.95)	14.3 kPa	0.90	0.98	0.77	0.99	0.397
FIB‐4	0.94 (0.88–0.97)	1.50	1.00	0.75	0.23	1.00	0.368
APRI	0.80 (0.72–0.87)	0.44	1.00	0.49	0.13	1.00	0.011
NFS	0.92 (0.85–0.96)	−1.08	0.80	0.86	0.30	0.98	0.270
≥ S1	*n* = 134						
MAP by AI‐POC‐TE	0.92 (0.86–0.96)	244 dB/m	0.94	0.80	0.98	0.50	—
CAP by conventional TE*	0.86 (0.79–0.91)	237 dB/m	0.74	0.90	0.99	0.21	0.250
HIS	0.84 (0.77–0.90)	33.3	0.85	0.80	0.98	0.29	0.342
≥ S2	*n* = 134						
MAP by AI‐POC‐TE	0.70 (0.62–0.78)	278 dB/m	0.82	0.53	0.73	0.65	—
CAP by conventional TE*	0.72 (0.64–0.80)	261 dB/m	0.75	0.64	0.77	0.62	0.520
HSI	0.68 (0.59–0.76)	38.9	0.62	0.68	0.75	0.53	0.692
S3	*n* = 134						
MAP by AI‐POC‐TE	0.75 (0.67–0.82)	294 dB/m	1.00	0.53	0.36	1.00	—
CAP by conventional TE*	0.75 (0.67–0.82)	280 dB/m	0.90	0.59	0.37	0.96	0.990
HSI	0.68 (0.59–0.76)	40.3	0.66	0.65	0.33	0.88	0.290

*Note:* Due to missing serological data, the head‐to‐head comparison analyses were performed on 132 and 134 patients for the fibrosis and steatosis category, respectively.

MAP had an AUROC of 0.92 for classifying any steatosis from no steatosis (S1–S3 vs. S0), which was nominally higher than CAP (0.86, no statistical difference by DeLong's test *p* = 0.25). On the other hand, MAP and CAP exhibited similar AUROCs in the diagnosis of ≥S2 (0.70 vs. 0.72, *p* = 0.52) and S3 (0.75 for both, *p* = 0.99). Performance of MAP was also compared with HSI in a subset of patients (*n* = 134, due to 2 missing serologic data). Although not statistically different (DeLong's test, *p* > 0.05 for all), both MAP and CAP had nominally higher AUROCs than HIS and were shown to be superior in determining different steatosis grades. Subgroup analyses (Table S2) confirmed that the AUROCs of AI‐POC‐TE remained comparable to conventional TE across the MASLD and CHB subgroups.

### 3.3 Correlation Analysis Between Conventional TE and AI‐POC‐TE

**FIGURE 3:**
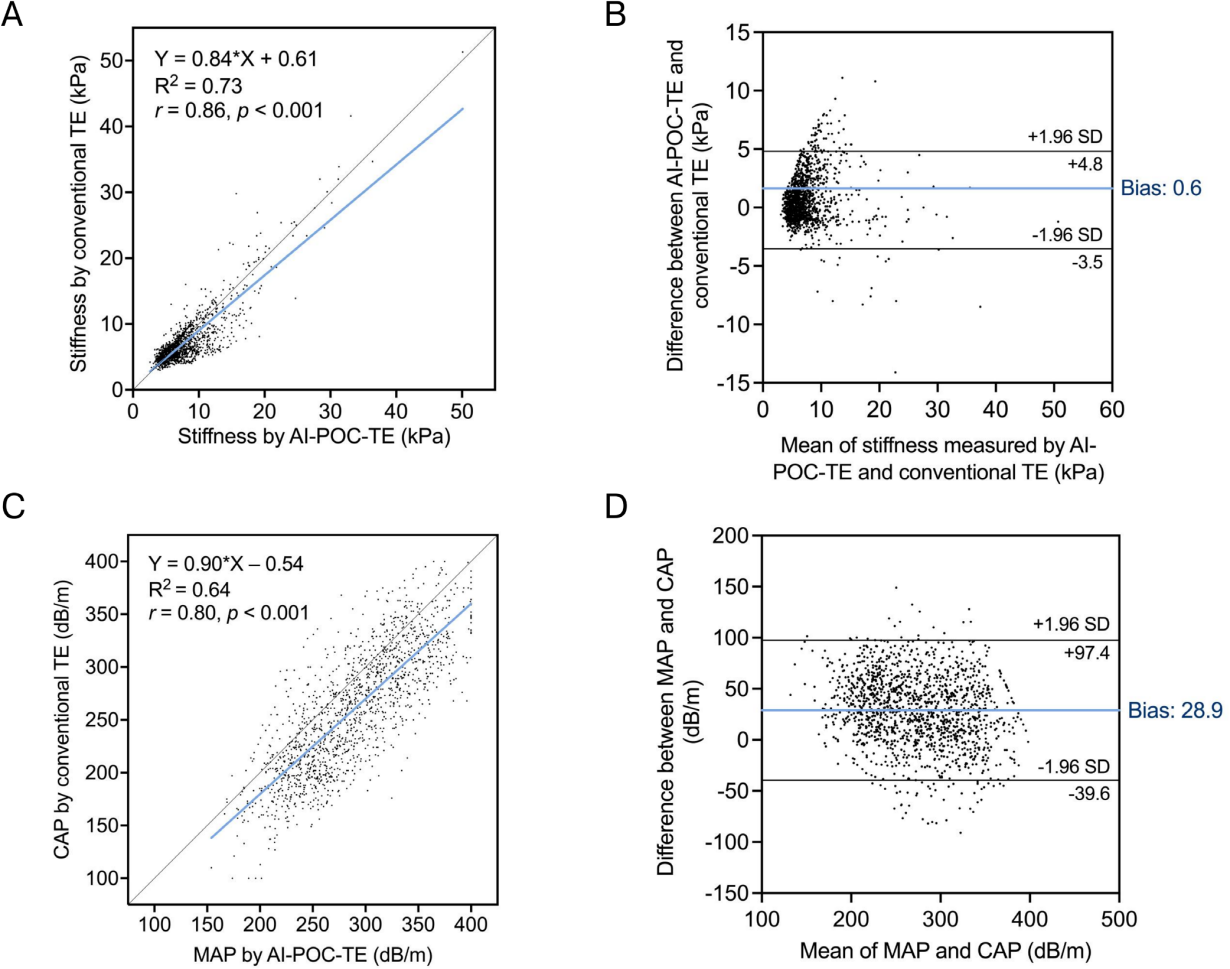
Comparison of LSM obtained from conventional TE and AI‐POC‐TE: (A) Scatterplot with Pearson correlation and linear regression and (B) Bland–Altman plot for 1455 participants; comparison of CAP from conventional TE and MAP from AI‐POC‐TE: (C) Scatterplot with Pearson correlation and linear regression and (D) Bland–Altman plot for 1455 participants.

LSMs with both TE techniques were obtained in 1455 patients of the *paired TE* cohort. As shown in Figure 3A, liver stiffness as assessed by the LSM and USG‐LSM methods was highly correlated and the linearity was strong (*r* = 0.86, *R* ^2^ = 0.73). This relationship was confirmed in a Bland–Altman plot (Figure 3B), demonstrating minimal bias of 0.6 kPa and limits of agreement (LoA) within ±5 kPa. Variability between LSMs, as indicated by the IQR/median ratio, was significantly higher in AI‐POC‐TE than conventional TE (median IQR/median value: 21 vs. 12%; *p* < 0.001). We further reported the influence of variability on diagnostic performance (see Table S3 for results).

Figure 3C,D illustrate the results of the corresponding 1455 paired CAP and MAP measurements. Similarly, MAP is moderately correlated to and linear with CAP (*r* = 0.80, R^2^ = 0.64). The mean bias was 29 dB/m with 95% LoA between −40 to 97 dB/m. As a reliability indicator of attenuation measurement, the IQR/median was not significantly different between CAP and MAP (median IQR/median value: 10% for both; *p* = 0.12). An additional exploratory analysis examining the influence of overweight and obesity (i.e., BMI ≥ 25 kg/m^2^) on the correlation between conventional TE and AI‐POC‐TE is illustrated in Figure S3 and Table S4. The correlation between techniques was comparable among patients with BMI < 25 and ≥ 25 kg/m^2^, for both LSMs (*r* = 0.89 vs. 0.83) and attenuation measurements (*r* = 0.80 vs. 0.73).

### 3.4 Multiclass Classification Concordance

Crosstabulation analyses showed substantial stage‐by‐stage agreement between techniques and with histologic reference, and the results are presented in Figures S4–S6.

### 3.5 Effect of Repetition Measurement on Diagnostic Performance

We generated a series of median sets derived from varying sample sizes (i.e., medians of 1 to all 10 repetition measurements). This analysis focused on five key histologic endpoints, and applied two mathematical approaches for median derivation. Table 4 details the results from the sequential selection method, taking the median of all 10 measurements as the reference. For LSM, the ICCs between the median of the first 1 through 9 measurements and the reference median of 10 measurements ranged from 0.90 to 0.99. The corresponding Spearman's correlation coefficients were all above 0.9, except for the 1‐measurement subset (*r*_s_ = 0.86). Bland–Altman analyses showed that both the systematic biases against the reference median and its 95% LoA range decreased progressively with an increasing number of measurements. The AUROC for diagnosing ≥ F2 fibrosis was highest (0.79, 95% CI: 0.71–0.86) when using the 10‐measurement median subset. In comparison, the AUROC significantly decreased to 0.74 when only a single measurement was used (DeLong's test, *p* = 0.03). A progressive decline in AUROC was observed as the number of measurements decreased from 9 to 2. The Wilcoxon tests further indicated significant differences between the medians derived from the first 1 and 2 measurements vs. the reference median (*p* = 0.01 and 0.03, respectively). By contrast, accuracy for cirrhosis remained stable across different measurement numbers, with AUROCs ranging from 0.93 to 0.97.

**TABLE 4:** Comparison of diagnostic accuracy and agreement metrics among the medians derived from different numbers of USG‐LSM and MAP measurements, using the sequential selection method.

Number of repetitions	First 1	First 2	First 3	First 4	First 5	First 6	First 7	First 8	First 9	10^a^
USG‐LSM for ≥ F2										
AUROC	0.739 [0.66, 0.81]	0.761 [0.68, 0.83]	0.777 [0.70, 0.84]	0.783 [0.71, 0.85]	0.781 [0.70, 0.85]	0.781 [0.70, 0.85]	0.786 [0.71, 0.85]	0.785 [0.71, 0.85]	0.784 [0.71, 0.85]	0.790 [0.71, 0.86]
DeLong's test, *p*‐value^b^	*0.030* ^c^	0.117	0.419	0.606	0.391	0.301	0.578	0.334	0.164	—
USG‐LSM for F4										
AUROC	0.925 [0.87, 0.96]	0.932 [0.88, 0.97]	0.948 [0.90, 0.98]	0.957 [0.91, 0.98]	0.959 [0.91, 0.99]	0.966 [0.92, 0.99]	0.970 [0.93, 0.99]	0.967 [0.92, 0.99]	0.971 [0.93, 0.99]	0.969 [0.93, 0.99]
DeLong's test, *p*‐value^b^	0.133	0.087	0.161	0.156	0.269	0.611	0.802	0.662	0.382	—
USG‐LSM for all stages F0–F4										
Bias [95% LoA]^d^	0.74 [−4.6, 6.1]	0.47 [−3.6, 4.5]	0.21 [−3.1, 3.5]	0.08 [−2.4, 2.5]	< 0.01 [−1.9, 1.9]	< 0.01 [−1.6, 1.6]	−0.01 [−1.2, 1.2]	0.05 [−0.8, 0.9]	−0.01 [−0.7, 0.7]	—
ICC^e^	0.90	0.94	0.96	0.98	0.99	0.99	> 0.99	> 0.99	> 0.99	—
Spearman's *r*_*s*_ ^e^	0.86	0.91	0.95	0.95	0.97	0.98	0.99	0.99	> 0.99	—
Wilcoxon test, *p*‐value^b^	*0.01*	*0.03*	> 0.05	> 0.05	> 0.05	> 0.05	> 0.05	> 0.05	> 0.05	—
Average IQR/median	0%	12%	18%	21%	21%	22%	23%	23%	24%	31%
MAP for ≥ S1										
AUROC	0.900 [0.84, 0.95]	0.909 [0.85, 0.95]	0.909 [0.85, 0.95]	0.921 [0.86, 0.96]	0.930 [0.87, 0.97]	0.935 [0.88, 0.97]	0.937 [0.88, 0.97]	0.932 [0.88, 0.97]	0.924 [0.87, 0.96]	0.922 [0.86, 0.96]
DeLong's test, *p*‐value^b^	0.580	0.674	0.640	0.977	0.715	0.427	0.349	0.300	0.652	—
MAP for ≥ S2										
AUROC	0.670 [0.58, 0.75]	0.657 [0.57, 0.74]	0.664 [0.58, 0.74]	0.681 [0.60, 0.76]	0.687 [0.60, 0.76]	0.699 [0.62, 0.78]	0.705 [0.62, 0.78]	0.709 [0.63, 0.78]	0.702 [0.62, 0.78]	0.697 [0.61, 0.77]
DeLong's test, *p*‐value^b^	0.303	*0.044* ^c^	0.055	0.245	0.396	0.851	0.335	0.057	0.235	—
MAP for S3										
AUROC	0.727 [0.64, 0.80]	0.734 [0.65, 0.81]	0.747 [0.67, 0.82]	0.740 [0.66, 0.81]	0.741 [0.66, 0.81]	0.750 [0.69, 0.82]	0.757 [0.68, 0.83]	0.763 [0.68, 0.83]	0.752 [0.67, 0.82]	0.758 [0.68, 0.83]
DeLong's test, *p*‐value^b^	0.272	0.324	0.574	0.217	0.186	0.450	0.818	0.444	0.233	—
MAP for all grades S0–S3										
Bias [95% LoA]^d^	0.40 [−55, 55]	1.44 [−37, 40]	0.07 [−35, 35]	1.37 [−28, 31]	0.96 [−23, 25]	0.09 [−17, 18]	−0.09 [−15, 15]	0.15 [−11, 11]	−0.45 [−8, 7]	—
ICC^e^	0.87	0.93	0.94	0.96	0.97	0.98	0.99	0.99	> 0.99	—
Spearman's *r*_*s*_ ^e^	0.87	0.92	0.94	0.96	0.97	0.98	0.99	0.99	> 0.99	—
Wilcoxon test, *p*‐value^b^	> 0.05	> 0.05	> 0.05	> 0.05	> 0.05	> 0.05	> 0.05	> 0.05	> 0.05	—
Average IQR/median	0%	4%	7%	8%	8%	8%	8%	8%	9%	9%

*Note:* Italic values indicate statistically significant results (*p* < 0.05).

Abbreviations: AI‐POC‐TE, artificial intelligence‐enabled point‐of‐care transient elastography; AUROC, area under the receiver operating characteristics curve; ICC, intraclass correlation coefficient; LoA, limits of agreement; MAP, multi‐domain attenuation parameter; *r*_*s*_, Spearman's rank correlation coefficient; USG‐LSM, ultrasonography‐guided liver stiffness measurement.

^a^ The median of 10 measurements serves as the reference in terms of ICC, correlation, Wilcoxon test, Delong's test and Bland–Altman analyses.

^b^ Difference between the medians of all 10 measurements vs. the first one measurement through first nine measurements.

^c^ AUROC comparison with the reference median subset indicates a statistical significance *p* < 0.05 according to DeLong's test.

^d^ Mean difference with 95% limits of agreement (LoA), based on Bland–Altman analysis, quantifies the agreement against the reference median derived from 10 measurements.

^e^ Association between the medians of all 10 measurements vs. the first measurement through the first nine measurements.

For MAP, both the ICC and Spearman correlation relative to the reference median increased progressively as the median included more measurements, from 1 up to 9. Accuracy was generally lower with fewer MAP measurements. Notably, the highest accuracy for each steatosis category was achieved using the median of 7 or 8 measurements, rather than the full set of ten. The AUROCs for severe steatosis were consistent across the medians of 1 to 10 measurements (0.73–0.76).

Similar patterns were observed under the random selection method (Table 5). Statistically significant differences were found in the AUROCs between the medians of 1–3 randomly selected measurements and that of 10 measurements for diagnosing ≥ F2, F4, ≥ S1 and ≥ S2 (DeLong's test, *p* < 0.05). In summary, these data generally suggest lower diagnostic performance with fewer than 4 measurements.

**TABLE 5:** Comparison of diagnostic accuracy and agreement metrics among the medians derived from different numbers of USG‐LSM and MAP measurements, using the random selection method.

Number of repetitions	Random 1	Random 2	Random 3	Random 4	Random 5	Random 6	Random 7	Random 8	Random 9	10^a^
USG‐LSM for ≥ F2										
AUROC	0.738 [0.66, 0.81]	0.758 [0.68, 0.83]	0.761 [0.68, 0.83]	0.773 [0.69, 0.84]	0.776 [0.70, 0.84]	0.788 [0.70, 0.85]	0.786 [0.71, 0.85]	0.791 [0.71, 0.86]	0.792 [0.72, 0.86]	0.790 [0.71, 0.86]
DeLong's test, *p*‐value^b^	*0.012* ^c^	*0.028* ^c^	*0.041* ^c^	0.132	0.155	0.216	0.447	0.946	0.727	—
USG‐LSM for F4										
AUROC	0.930 [0.87, 0.97]	0.937 [0.88, 0.97]	0.948 [0.90, 0.98]	0.961 [0.91, 0.99]	0.969 [0.93, 0.99]	0.970 [0.93, 0.99]	0.970 [0.93, 0.99]	0.970 [0.93, 0.99]	0.970 [0.93, 0.99]	0.969 [0.93, 0.99]
DeLong's test, *p*‐value^d^	0.059	*0.027* ^c^	*0.039* ^c^	0.117	1.000	0.756	0.768	0.503	0.590	—
USG‐LSM for all stages F0–F4										
Bias [95% LoA]^e^	−0.12 [−4.7, 4.4]	0.10 [−3.3, 3.5]	−0.03 [−2.6, 2.5]	−0.08 [−2.0, 1.9]	−0.12 [−1.6, 1.4]	−0.02 [−1.4, 1.4]	−0.06 [−1.1, 1.0]	−0.01 [−1.0, 1.0]	0.02 [−0.7, 0.7]	—
ICC^d^	0.93	0.96	0.98	0.99	0.99	0.99	> 0.99	> 0.99	> 0.99	—
Spearman's *r*_*s*_ ^d^	0.90	0.95	0.95	0.97	0.97	0.98	0.99	0.99	> 0.99	—
Wilcoxon test, *p*‐value^b^	> 0.05	> 0.05	> 0.05	> 0.05	*0.02*	> 0.05	> 0.05	> 0.05	> 0.05	—
Average IQR/median	0%	11%	18%	21%	21%	23%	23%	23%	23%	31%
MAP for ≥ S1										
AUROC	0.775 [0.70, 0.84]	0.876 [0.81, 0.93]	0.900 [0.84, 0.95]	0.925 [0.87, 0.96]	0.921 [0.86, 0.96]	0.918 [0.86, 0.96]	0.919 [0.86, 0.96]	0.918 [0.86, 0.96]	0.925 [0.87, 0.96]	0.922 [0.86, 0.96]
DeLong's test, *p*‐value^b^	*0.009* ^c^	*0.034* ^c^	0.129	0.742	0.937	0.292	0.602	0.213	0.468	—
MAP for ≥ S2										
AUROC	0.613 [0.53, 0.70]	0.666 [0.58, 0.74]	0.703 [0.62, 0.78]	0.714 [0.63, 0.79]	0.704 [0.62, 0.78]	0.708 [0.62, 0.78]	0.705 [0.62, 0.78]	0.702 [0.62, 0.78]	0.698 [0.61, 0.77]	0.697 [0.61, 0.77]
DeLong's test, *p*‐value^b^	*0.001* ^c^	0.097	0.681	0.139	0.551	0.221	0.340	0.374	0.938	—
MAP for S3										
AUROC	0.709 [0.62, 0.78]	0.739 [0.66, 0.81]	0.772 [0.69, 0.84]	0.767 [0.68, 0.84]	0.756 [0.68, 0.83]	0.756 [0.68, 0.83]	0.755 [0.67, 0.83]	0.759 [0.68, 0.83]	0.751 [0.67, 0.82]	0.758 [0.68, 0.83]
DeLong's test, *p*‐value^b^	0.109	0.488	0.455	0.498	0.861	0.822	0.800	0.949	0.133	—
MAP for all grades S0–S3										
Bias [95% LoA]^e^	2.53 [−53, 58]	0.23 [−39, 39]	−0.66 [−35, 33]	0.20 [−25, 25]	0.17 [−22, 22]	0.11 [−16, 16]	−1.05 [−17, 14]	−0.66 [−12, 11]	−0.35 [−8, 7]	—
ICC^d^	0.85	0.92	0.94	0.97	0.98	0.99	0.99	0.99	> 0.99	—
Spearman's *r*_*s*_ ^d^	0.85	0.91	0.95	0.97	0.97	0.98	0.99	0.99	> 0.99	—
Wilcoxon test, *p*‐value^b^	> 0.05	> 0.05	> 0.05	> 0.05	> 0.05	> 0.05	> 0.05	> 0.05	> 0.05	—
Average IQR/median	0%	5%	7%	8%	9%	9%	9%	9%	9%	9%

*Note:* Italic values indicate statistically significant results (*p* < 0.05).

Abbreviations: AI‐POC‐TE, artificial intelligence‐enabled point‐of‐care transient elastography; AUROC, area under the receiver operating characteristics curve; ICC, intraclass correlation coefficient; LoA, limits of agreement.; MAP, multi‐domain attenuation parameter; *r*_*s*_, Spearman's rank correlation coefficient; USG‐LSM, ultrasonography‐guided liver stiffness measurement.

^a^ The median of 10 measurements serves as the reference in terms of ICC, correlation, Wilcoxon test, Delong's test and Bland–Altman analyses.

^b^ Difference between the medians of all 10 measurements vs. the random one measurement through random nine measurements.

^c^ AUROC comparison with the reference median subset indicates a statistical significance *p* < 0.05 according to DeLong's test.

^d^ Association between the medians of all 10 measurements vs. the random one measurement through random nine measurements.

^e^ Mean difference with 95% limits of agreement (LoA), based on Bland–Altman analysis, quantifies the agreement against the reference median derived from 10 measurements.

## 4 Discussion

The role of TE in the monitoring of liver diseases is well established; however, technology‐related limitations persist regarding its accessibility and utility, such as large footprint, inadequate imaging guidance, and traditional algorithmic frameworks. Addressing these issues requires bioengineering innovation. This is our motivation to develop a new form of TE—AI‐POC‐TE which integrates AI‐driven analysis and B‐mode as visual guidance for the point‐of‐care assessment. In the prospective study, we introduce this new TE iteration and report its diagnostic performance compared to liver histology. Furthermore, we investigated the ideal number of LSM and MAP from which to obtain repetition measurements. Our findings demonstrate three major contributions to the field: (1) AI‐POC‐TE achieved robust accuracy for staging fibrosis and steatosis, with AUROCs comparable to conventional TE and exceeding other established serologic tests; (2) the MAP, a novel ultrasound attenuation computation method in AI‐POC‐TE, provided methodological advantages over CAP for steatosis detection; (3) the number of measurements required to maintain accuracy is only 4, which is fewer than the conventionally mandated 10 and indicates the potential for workflow optimization.

### 4.1 Fibrosis and Steatosis Quantification Using AI‐POC‐TE

AI‐POC‐TE showed good performance for assessing dichotomized stages of fibrosis, with AUROC values ranging from 0.79 to 0.96. While diagnostic accuracy was modest for early stages, the results were broadly consistent with prior reports on conventional TE in biopsy‐controlled cohorts [25, 26]. This suggests that AI‐POC‐TE maintains the established strengths of TE in fibrosis diagnosis, while miniaturizing the hardware, introducing a wireless workflow, and providing B‐mode guidance that may reduce the risk of off‐target measurement and sampling error. We also confirmed a strong correlation between liver stiffness measured using both TE techniques (*r* = 0.86) in nearly 1500 patients. On the other hand, the correlation between techniques was less pronounced for attenuation measurements (*r* = 0.80) than for LSMs. This likely reflected methodological differences in the attenuation computation process, whereas liver stiffness was derived based on the same physical principle. Indeed, MAP nominally improved the discrimination of any steatosis compared with CAP (AUROC: 0.92 vs. 0.86), highlighting the potential of spatially averaged, multi‐domain analysis for more robust fat quantification. However, MAP yielded suboptimal ability to distinguish moderate‐to‐severe steatosis, with the small cut‐off difference between S2 and S3. This phenomenon has also been reported for other ultrasound attenuation‐based methods in literature, including CAP [7, 25] and attenuation imaging [27]. Additionally, AI‐POC‐TE outperformed the studied serum biomarkers in assessing fibrosis and steatosis, a finding that confirms prior reports from conventional TE [7, 25].

### 4.2 Clinical Implications of POCUS and AI Integration

AI‐driven approaches show promise in CLD management, but mainly for diagnosis and prediction tasks [17, 18]. Only a few studies have by far applied AI in the context of liver elastography [19]. Furthermore, there is a paucity of literature reporting adding AI to TE. In this study, we made the first attempt to utilize a range of deep learning algorithms to optimize the entire TE workflow: a segmentation model selected the appropriate measurement mode by adapting acquisition parameters to abdominal wall thickness; a binary classifier ensured elastogram quality; a line‐detection model enhanced shear wave trajectory detection; and another segmentation model identified parenchymal regions to improve attenuation computation. Future studies will explore radiomics of B‐mode data, which is concurrently available, and develop stacked ensemble learning models for assessing MASLD spectrum, particularly MASH. Another direction is to enhance workflow efficiency and support novice operators. Specific AI applications include elastogram quality enhancement through generative models, as well as scan guide based on B‐mode liver segmentation or reinforcement learning.

With the recent approval of resmetirom [28] and semaglutide [29] as treatment for MASH with fibrosis, and with non‐invasive markers being considered by regulatory authorities as non‐histological surrogate clinical endpoints, the clinical demand for TE examination is anticipated to rise [10]. Indeed, the incorporation of POCUS into liver elastography or quantitative fat analysis has been recently explored for improved technology accessibility [30, 31, 32]. To our best knowledge, this study employed the most compact TE system ever reported, which may facilitate the widespread uptake in both clinical and public health settings. It will be critical for the roll‐out of the technique in resource‐limited regions.

### 4.3 How Many Measurements Are Really Necessary?

Another novel contribution of this study was quantifying the trade‐off between measurement number and diagnostic accuracy. Current consensus requires 10 measurements for TE [6]; but it is somewhat surprising that, what constitutes an adequate number of TE has never been examined empirically and is largely a matter of opinion. This opinion is taken for granted in various clinical practice guidelines and large studies. In contrast, pSWE and 2D‐SWE acquisition protocols have been well investigated, and their findings continuously inform guideline updates [6, 33, 34, 35]. We speculated that conventional TE, without B‐mode guidance to ensure continuous targeting of the liver parenchyma, may explain such a difference. Thus, we employed B‐mode guided TE to bridge the knowledge gap. Results suggest that fewer than 10 measurements, selected either randomly or in sequence, had a limited effect on the performance of both LSM and MAP, potentially saving clinic time. Even with 1 available acquisition, whether random or serial, the AUROCs for diagnosing fibrosis and steatosis were acceptable; nevertheless, at least 4 acquisitions were generally required. This finding aligns with the recommended range of 3–5 acquisitions for SWE‐based techniques [6, 34, 35]. We speculated that this might also be the case for conventional TE (Fibroscan), as the comparable measurement results (i.e., median value and variability) between two methods were demonstrated in this study. Yet one study alone is rarely sufficient evidence to justify a change in clinical practice. Larger external validation cohorts are warranted to examine our empiric data.

### 4.4 Strengths and Limitations

There were several notable strengths of the current study. The time interval between biopsy and TE was limited to the same day, minimizing temporal bias and generally shorter than in most previous biopsy‐controlled studies. By directly comparing AI‐POC‐TE with well‐validated conventional TE in the same patient group, the study allowed a rigorous, unbiased evaluation of system performance. The cut‐offs for both conventional TE and AI‐POC‐TE derived in this study are broadly in keeping with those reported in meta‐analyses of CLD patients [26, 36]. We first defined a minimally sufficient number of measurements, thereby providing evidence‐based recommendations for refining the existing TE protocol. Another novelty is that, for the first time, AI was incorporated into TE as part of data processing and analytics.

Some limitations are acknowledged. First, our subgroup analyses (Table S2) demonstrated robust accuracy for both MASLD and CHB, but should be interpreted cautiously due to the limited sample size of CHB patients. The diagnostic cut‐offs require future validation in larger, aetiology‐specific international populations. Second, we investigated only the diagnostic accuracy of B‐mode guided TE, although additional performance characteristics, including reliability, repeatability, construct validity as well as examination time against other elastography techniques, also merit mention. However, some of these, such as operator ICC and correlation with 2D‐SWE, have been reported elsewhere [11, 12, 20]. Third, because this was a single‐centre study in a highly specialized tertiary care setting, the generalizability of its findings to other clinical settings is unknown. Extrapolation to primary care (e.g., community screening) is promising but needs further confirmation.

## 5 Conclusions

The newly developed and validated AI‐POC‐TE can accurately assess histology‐confirmed fibrosis and steatosis, with performance comparable to conventional TE. Another main finding suggests that, at least in a B‐mode image guided setting, surprisingly four measurements were sufficient for diagnostic purposes. AI‐driven analysis was demonstrably feasible in this study, but its performance in pediatric and obese populations warrants further investigation. Future research should involve multi‐centre cohorts and establish cut‐offs for different aetiologies.

## Author Contributions

Zi‐Hao Huang and Chen‐Hui Ye are considered joint first authors. Yong‐Ping Zheng and Ming‐Hua Zheng are considered joint senior authors. All authors approved the final version of the article. Conceptualization, study design: Zi‐Hao Huang, Chong‐Lin Wu, Wan‐Rui Li, Chen‐Hui Ye, Yong‐Ping Zheng, Ming‐Hua Zheng. Data acquisition, data analyses, statistical analyses: Zi‐Hao Huang, Li‐You Lian, Chong‐Lin Wu, Miao‐Qin Deng, Chen‐Hui Ye, Chen‐Xiao Huang, Yi‐Xuan Wei, Ying‐Ying Cao, Xiao‐Na Shen, Yi‐Wei Lin, Sui‐Dan Chen. Data interpretation, writing of the manuscript: Zi‐Hao Huang, Chen‐Hui Ye, Wan‐Rui Li, Wai‐Kay Seto, Yong‐Ping Zheng, Ming‐Hua Zheng. Critical review and editing of the manuscript: Zi‐Hao Huang, Wai‐Kay Seto, Yong‐Ping Zheng, Ming‐Hua Zheng.

## Funding

This study was funded in part by The Hong Kong Polytechnic University (grant number: 4‐W410).

## Ethics Statement

Ethical approval was obtained from the local institutional review board (2016‐0246, 2018‐059).

## Consent

Written informed consent was obtained from all patients involved in the study.

## Conflicts of Interest

Wai‐Kay Seto received speaker's fees from Echosens and MSD, is an advisory board member and received speaker's fees from Abbott, received research funding via the University of Hong Kong from 89Bio, AstraZeneca, Boehringer Ingelheim, Pfizer, and Ribo Life Science, and is an advisory board member, received speaker's fees and research funding via the University of Hong Kong from Gilead Sciences. Yong‐Ping Zheng owns the patents related to the technology of B‐mode imaging guided transient elastography (CN101843501B, US8147410B2), which have been licensed to Eieling Technology Limited. He also served as an advisory board member for the company via his home university of the Hong Kong Polytechnic University. Ming‐Hua Zheng has received honoraria for lectures from AstraZeneca, Hisky Medical Technologies, and Novo Nordisk, and consulting fees from Boehringer Ingelheim. All other authors report no conflicts of interest.

## Supporting information


**Figure S1:** Flow diagram of participants;
**Figure S2:** Receiver‐operating characteristic curves of USG‐LSM and MAP measured by AI‐POC‐TE;
**Figure S3:** Scatterplots of conventional TE (Fibroscan) vs. AI‐POC‐TE according to BMI category;
**Figures S4–S6:** Classification concordance matrices between histology, conventional TE (Fibroscan), and AI‐POC‐TE under various criteria.
**Table S1:** Diagnostic test characteristics of conventional TE (Fibroscan) for hepatic fibrosis and steatosis.
**Tables S2–S4:** Comparative diagnostic accuracy of AI‐POC‐TE across different liver disease aetiologies, reliability criteria, and BMI categories.

## Data Availability

The data that support the findings of this study are available from the corresponding author upon reasonable request. The data are not publicly available due to privacy or ethical restrictions.

## References

[1] A. A. Mokdad , A. D. Lopez , S. Shahraz , et al., “Liver Cirrhosis Mortality in 187 Countries Between 1980 and 2010: A Systematic Analysis,” BMC Medicine 12, no. 1 (2014): 145, 10.1186/s12916-014-0145-y. 25242656 PMC4169640

[2] N. Noureddin , D. Q. Huang , R. Bettencourt , et al., “Natural History of Clinical Outcomes and Hepatic Decompensation in Metabolic Dysfunction‐Associated Steatotic Liver Disease,” Alimentary Pharmacology & Therapeutics 59, no. 12 (2024): 1521–1526, 10.1111/apt.17981. 38571305 PMC12020952

[3] G. Feng , L. Valenti , V. W.‐S. Wong , et al., “Recompensation in Cirrhosis: Unravelling the Evolving Natural History of Nonalcoholic Fatty Liver Disease,” Nature Reviews Gastroenterology & Hepatology 21, no. 1 (2024): 46–56, 10.1038/s41575-023-00846-4. 37798441

[4] R. P. Myers , A. Fong , and A. A. M. Shaheen , “Utilization Rates, Complications and Costs of Percutaneous Liver Biopsy: A Population‐Based Study Including 4275 Biopsies,” Liver International 28, no. 5 (2008): 705–712, 10.1111/j.1478-3231.2008.01691.x. 18433397

[5] L. Sandrin , B. Fourquet , J.‐M. Hasquenoph , et al., “Transient Elastography: A New Noninvasive Method for Assessment of Hepatic Fibrosis,” Ultrasound in Medicine & Biology 29, no. 12 (2003): 1705–1713, 10.1016/j.ultrasmedbio.2003.07.001. 14698338

[6] G. Ferraioli , R. G. Barr , A. Berzigotti , et al., “WFUMB Guideline/Guidance on Liver Multiparametric Ultrasound: Part 1. Update to 2018 Guidelines on Liver Ultrasound Elastography,” Ultrasound in Medicine & Biology 50, no. 8 (2024): 1071–1087, 10.1016/j.ultrasmedbio.2024.03.013. 38762390

[7] R. P. Myers , A. Pollett , R. Kirsch , et al., “Controlled Attenuation Parameter (CAP): A Noninvasive Method for the Detection of Hepatic Steatosis Based on Transient Elastography,” Liver International 32, no. 6 (2012): 902–910, 10.1111/j.1478-3231.2012.02781.x. 22435761

[8] K. Cusi , S. Isaacs , D. Barb , et al., “American Association of Clinical Endocrinology Clinical Practice Guideline for the Diagnosis and Management of Nonalcoholic Fatty Liver Disease in Primary Care and Endocrinology Clinical Settings: Co‐Sponsored by the American Association for the Study of Liver Diseases (AASLD),” Endocrine Practice 28, no. 5 (2022): 528–562, 10.1016/j.eprac.2022.03.010. 35569886

[9] M. E. Rinella , B. A. Neuschwander‐Tetri , M. S. Siddiqui , et al., “AASLD Practice Guidance on the Clinical Assessment and Management of Nonalcoholic Fatty Liver Disease,” Hepatology 77, no. 5 (2023): 1797–1835. 36727674 10.1097/HEP.0000000000000323 PMC10735173

[10] G. Feng , V. W.‐S. Wong , G. Targher , C. D. Byrne , and M.‐H. Zheng , “Non‐Invasive Tests of Fibrosis in the Management of MASLD: Revolutionising Diagnosis, Progression and Regression Monitoring,” Gut 74, no. 10 (2025): 1741–1750, 10.1136/gutjnl-2025-335542. 40675797

[11] Z.‐H. Huang , L.‐K. Wang , S.‐Y. Cai , et al., “Palm‐Sized Wireless Transient Elastography System With Real‐Time B‐Mode Ultrasound Imaging Guidance: Toward Point‐Of‐Care Liver Fibrosis Assessment,” Diagnostics (Basel) 14, no. 2 (2024): 189, 10.3390/diagnostics14020189. PMC11154523 38248066

[12] T.‐M. Mak , Y.‐P. Huang , and Y.‐P. Zheng , “Liver Fibrosis Assessment Using Transient Elastography Guided With Real‐Time B‐Mode Ultrasound Imaging: A Feasibility Study,” Ultrasound in Medicine & Biology 39, no. 6 (2013): 956–966, 10.1016/j.ultrasmedbio.2013.01.009. 23562022

[13] V. Y.‐F. Leung , J. Shen , V. W.‐S. Wong , et al., “Quantitative Elastography of Liver Fibrosis and Spleen Stiffness in Chronic Hepatitis B Carriers: Comparison of Shear‐Wave Elastography and Transient Elastography With Liver Biopsy Correlation,” Radiology 269, no. 3 (2013): 910–918, 10.1148/radiol.13130128. 23912619

[14] K. Patel and G. Sebastiani , “Limitations of Non‐Invasive Tests for Assessment of Liver Fibrosis,” JHEP Reports 2, no. 2 (2020): 100067, 10.1016/j.jhepr.2020.100067. 32118201 PMC7047178

[15] S. Audière , A. Labourdette , V. Miette , et al., “Improved Ultrasound Attenuation Measurement Method for the Non‐Invasive Evaluation of Hepatic Steatosis Using FibroScan,” Ultrasound in Medicine & Biology 47, no. 11 (2021): 3181–3195, 10.1016/j.ultrasmedbio.2021.07.007. 34373137

[16] Y. Fujiwara , H. Kuroda , T. Abe , et al., “The B‐Mode Image‐Guided Ultrasound Attenuation Parameter Accurately Detects Hepatic Steatosis in Chronic Liver Disease,” Ultrasound in Medicine & Biology 44, no. 11 (2018): 2223–2232, 10.1016/j.ultrasmedbio.2018.06.017. 30077415

[17] J. Yi , H. K. Kang , J.‐H. Kwon , et al., “Technology Trends and Applications of Deep Learning in Ultrasonography: Image Quality Enhancement, Diagnostic Support, and Improving Workflow Efficiency,” Ultrasonography (Seoul, Korea) 40, no. 1 (2021): 7–22, 10.14366/usg.20102. 33152846 PMC7758107

[18] H. Zhang , X. Wu , W. Ni , et al., “Application of Machine Learning and Deep Learning in Metabolic Dysfunction‐Associated Steatotic Liver Disease: A Systematic Review and Meta‐Analysis,” Journal of Advanced Research 22 (2025), 10.1016/j.jare.2025.08.042.40850679

[19] X.‐Y. Zhang , Q. Wei , G.‐G. Wu , et al., “Artificial Intelligence–Based Ultrasound Elastography for Disease Evaluation—A Narrative Review,” Frontiers in Oncology 13 (2023): 1197447, 10.3389/fonc.2023.1197447. 37333814 PMC10272784

[20] Z.‐H. Huang , M.‐Q. Deng , Y. Lin , C.‐H. Ye , M.‐H. Zheng , and Y.‐P. Zheng , “Body Posture Can Modulate Liver Stiffness Measured by Transient Elastography: A Prospective Observational Study,” BMC Gastroenterology 24, no. 1 (2024): 386, 10.1186/s12876-024-03473-8. 39482593 PMC11526721

[21] X. Wang , B. Liu , C. Wu , et al., “Shear Wave Trajectory Detection in Ultra‐Fast M‐Mode Images for Liver Fibrosis Assessment: A Deep Learning‐Based Line Detection Approach,” Ultrasonics 142 (2024): 107358, 10.1016/j.ultras.2024.107358. 38901149

[22] S. Woo , S. Debnath , R. Hu , et al., “ConvNeXt V2: Co‐Designing and Scaling ConvNets With Masked Autoencoders,” (2023), 10.48550/arxiv.2301.00808.

[23] J. Terven , D.‐M. Córdova‐Esparza , and J.‐A. Romero‐González , “A Comprehensive Review of YOLO Architectures in Computer Vision: From YOLOv1 to YOLOv8 and YOLO‐NAS,” Machine Learning and Knowledge Extraction 5, no. 4 (2023): 1680–1716, 10.3390/make5040083.

[24] C. Yu , C. Gao , J. Wang , G. Yu , C. Shen , and N. Sang , “BiSeNet V2: Bilateral Network With Guided Aggregation for Real‐Time Semantic Segmentation,” International Journal of Computer Vision 129, no. 11 (2021): 3051–3068, 10.1007/s11263-021-01515-2.

[25] P. J. Eddowes , M. Sasso , M. Allison , et al., “Accuracy of FibroScan Controlled Attenuation Parameter and Liver Stiffness Measurement in Assessing Steatosis and Fibrosis in Patients With Nonalcoholic Fatty Liver Disease,” Gastroenterology 156, no. 6 (2019): 1717–1730. 30689971 10.1053/j.gastro.2019.01.042

[26] E. A. Tsochatzis , K. S. Gurusamy , S. Ntaoula , E. Cholongitas , B. R. Davidson , and A. K. Burroughs , “Elastography for the Diagnosis of Severity of Fibrosis in Chronic Liver Disease: A Meta‐Analysis of Diagnostic Accuracy,” Journal of Hepatology 54, no. 4 (2011): 650–659, 10.1016/j.jhep.2010.07.033. 21146892

[27] T. Y. Huang , Z. Y. Li , J. Tian , et al., “Metabolic Dysfunction–Associated Steatotic Liver Disease at Quantitative US: International Prospective Study,” Radiology 316, no. 1 (2025): e242564, 10.1148/radiol.242564. 40662970

[28] S. A. Harrison , P. Bedossa , C. D. Guy , et al., “A Phase 3, Randomized, Controlled Trial of Resmetirom in NASH With Liver Fibrosis,” New England Journal of Medicine 390, no. 6 (2024): 497–509, 10.1056/NEJMoa2309000. 38324483

[29] A. J. Sanyal , P. N. Newsome , I. Kliers , et al., “Phase 3 Trial of Semaglutide in Metabolic Dysfunction–Associated Steatohepatitis,” New England Journal of Medicine 392, no. 21 (2025): 2089–2099, 10.1056/NEJMoa2413258. 40305708

[30] D. Tamayo‐Murillo , J. T. Weeks , C. A. Keller , et al., “Quantitative Liver Fat Assessment by Handheld Point‐Of‐Care Ultrasound: A Technical Implementation and Pilot Study in Adults,” Ultrasound in Medicine & Biology 51, no. 3 (2025): 475–483, 10.1016/j.ultrasmedbio.2024.11.005. 39690042 PMC12258410

[31] R. Loomba , A. Ramji , T. Hassanein , et al., “Velacur ACE Outperforms FibroScan CAP for Diagnosis of MASLD,” Hepatology Communications 8, no. 4 (2024): e0402, 10.1097/HC9.0000000000000402. 38517204 PMC10962894

[32] A. Besson , B. Hériard‐Dubreuil , J. Gay , A. Delamarre , J. Foucher , and C. Cohen‐Bacrie , “Quantitative Ultrasound for Steatosis Assessment Using Hepatoscope: Confounding Technical Factors,” WFUMB Ultrasound Open 2, no. 2 (2024): 100069, 10.1016/j.wfumbo.2024.100069.

[33] C. Fang , O. S. Jaffer , G. T. Yusuf , et al., “Reducing the Number of Measurements in Liver Point Shear‐Wave Elastography: Factors That Influence the Number and Reliability of Measurements in Assessment of Liver Fibrosis in Clinical Practice,” Radiology 287, no. 3 (2018): 844–852, 10.1148/radiol.2018172104. 29514018

[34] S. H. Choi , W. K. Jeong , Y. Kim , et al., “How Many Times Should We Repeat Measuring Liver Stiffness Using Shear Wave Elastography?: 5‐Repetition Versus 10‐Repetition Protocols,” Ultrasonics 72 (2016): 158–164, 10.1016/j.ultras.2016.08.005. 27529140

[35] M. Ronot , G. Ferraioli , H.‐P. Müller , et al., “Comparison of Liver Stiffness Measurements by a 2D‐Shear Wave Technique and Transient Elastography: Results From a European Prospective Multi‐Centre Study,” European Radiology 31, no. 3 (2021): 1578–1587, 10.1007/s00330-020-07212-x. 32902745

[36] T. Karlas , D. Petroff , M. Sasso , et al., “Individual Patient Data Meta‐Analysis of Controlled Attenuation Parameter (CAP) Technology for Assessing Steatosis,” Journal of Hepatology 66, no. 5 (2017): 1022–1030, 10.1016/j.jhep.2016.12.022. 28039099

