# Supplementary material for: Identification of Hepatic Fibrosis and Steatosis via A Point‐of‐Care Transient Elastography System With Integrated AI
Source: Liver Int. 2026 Apr 8;46(5):e70634. doi: 10.1111/liv.70634 (PMC13058509; doi:10.1111/liv.70634)
Supplement: Supplementary file 1 — Figure S1: Flow diagram of participants; Figure S2: Receiver‐operating characteristic curves of USG‐LSM and MAP measured by AI‐POC‐TE; Figure S3: Scatterplots of conventional TE (Fibroscan) vs. AI‐POC‐TE according to BMI category; Figures S4–S6: Classification concordance matrices between histology, conventional TE (Fibroscan), and AI‐POC‐TE under various criteria. Table S1: Diagnostic test characteristics of conventional TE (Fibroscan) for hepatic fibrosis and steatosis. Tables S2–S4: Comparative diagnostic accuracy of AI‐POC‐TE across different liver disease aetiologies, reliability criteria, and BMI categories. [file LIV-46-0-s001.docx]

# Supplementary Material

### ***Supplementary Figures***

**Supplementary Figure 1.** **Flow diagram of participants.**

**
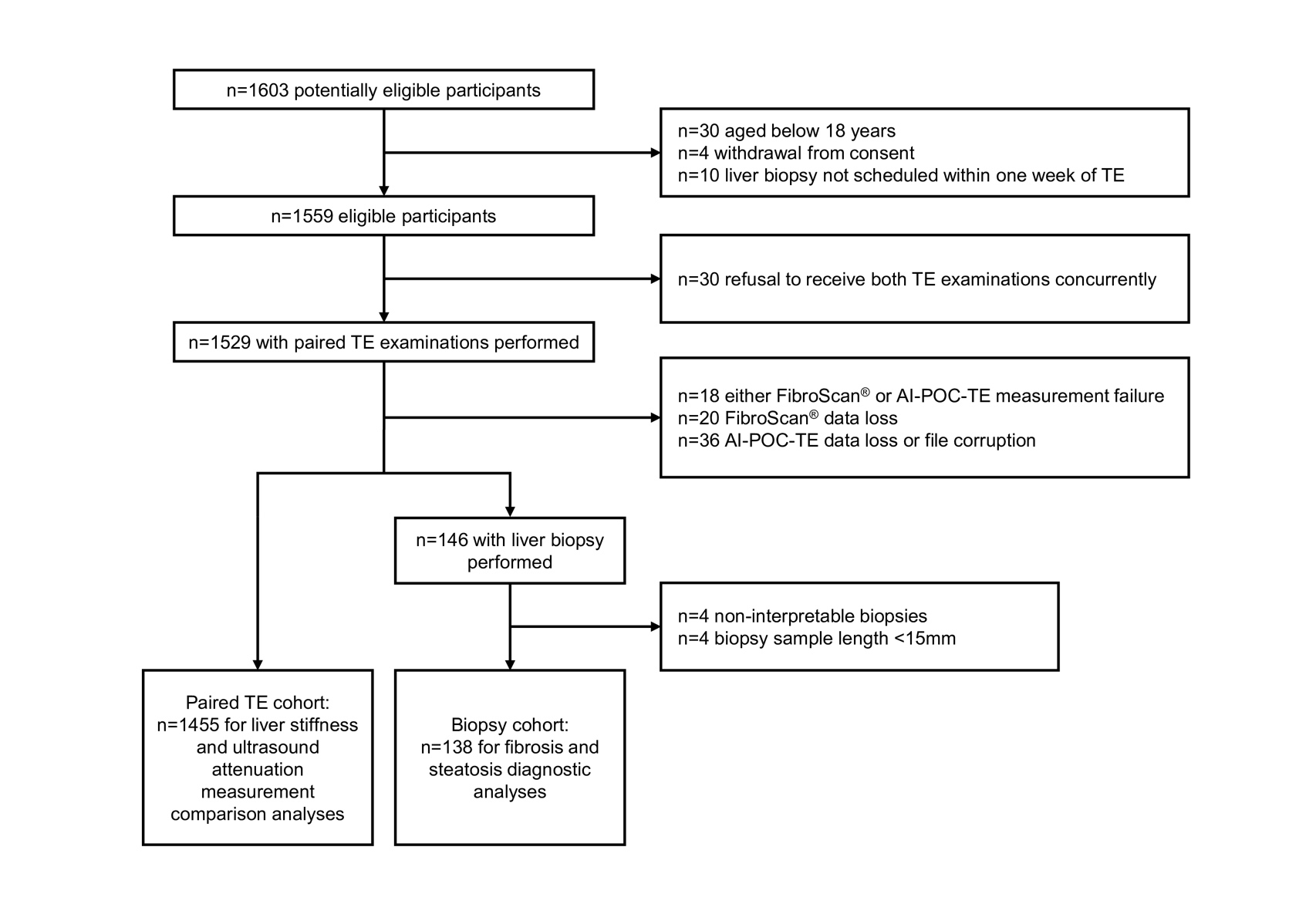
**

**Supplementary Figure 2.** **Receiver-operating characteristic curves of (A) USG-LSM and (B) MAP measured by AI-POC-TE for differentiating between dichotomized fibrosis stages and steatosis grades.**

1.
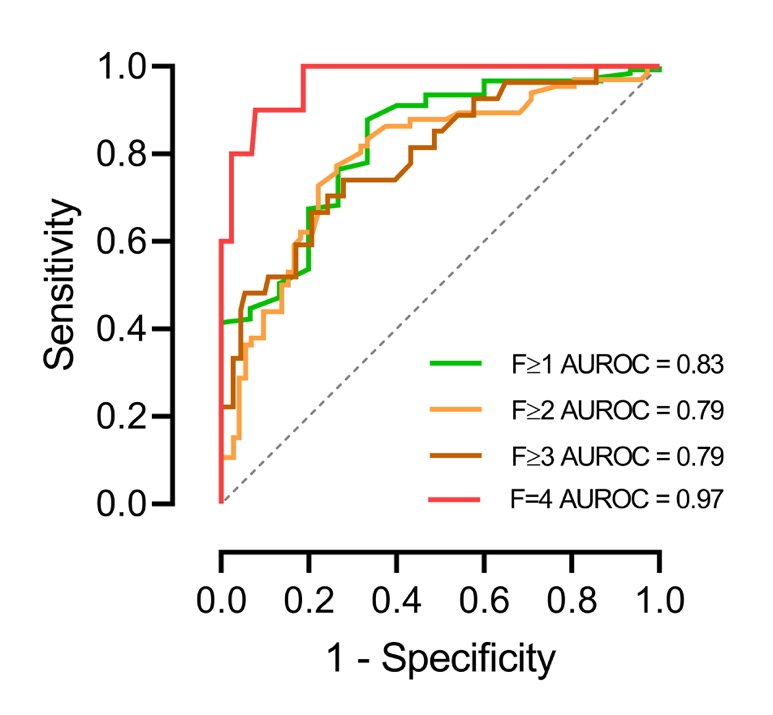

2.
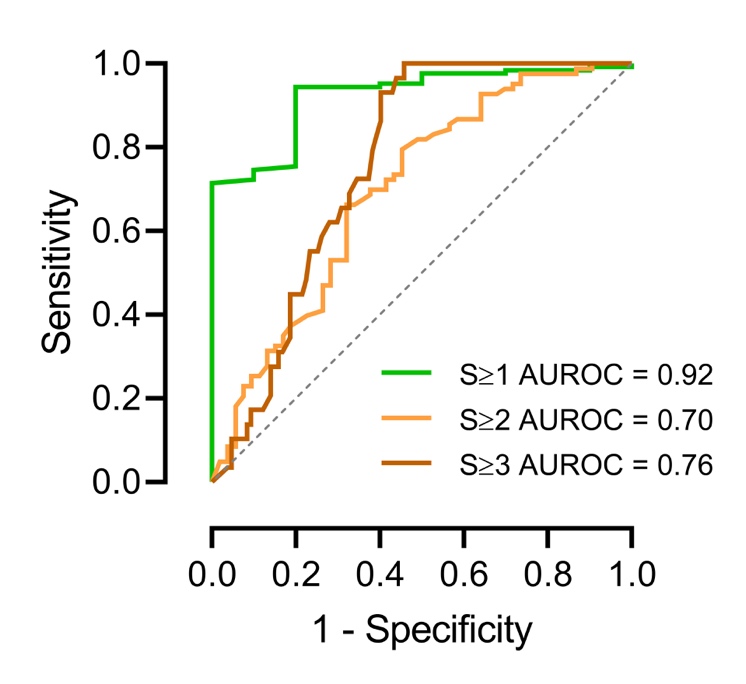


**Supplementary Figure 3. Scatterplots of (A) liver stiffness measured by conventional TE vs. AI-POC-TE; and (B) CAP measured by conventional TE vs. MAP measured by AI-POC-TE (grey and blue circles indicate the patients with BMI ≥25 kg/m2 and the controls, respectively).**

(A)
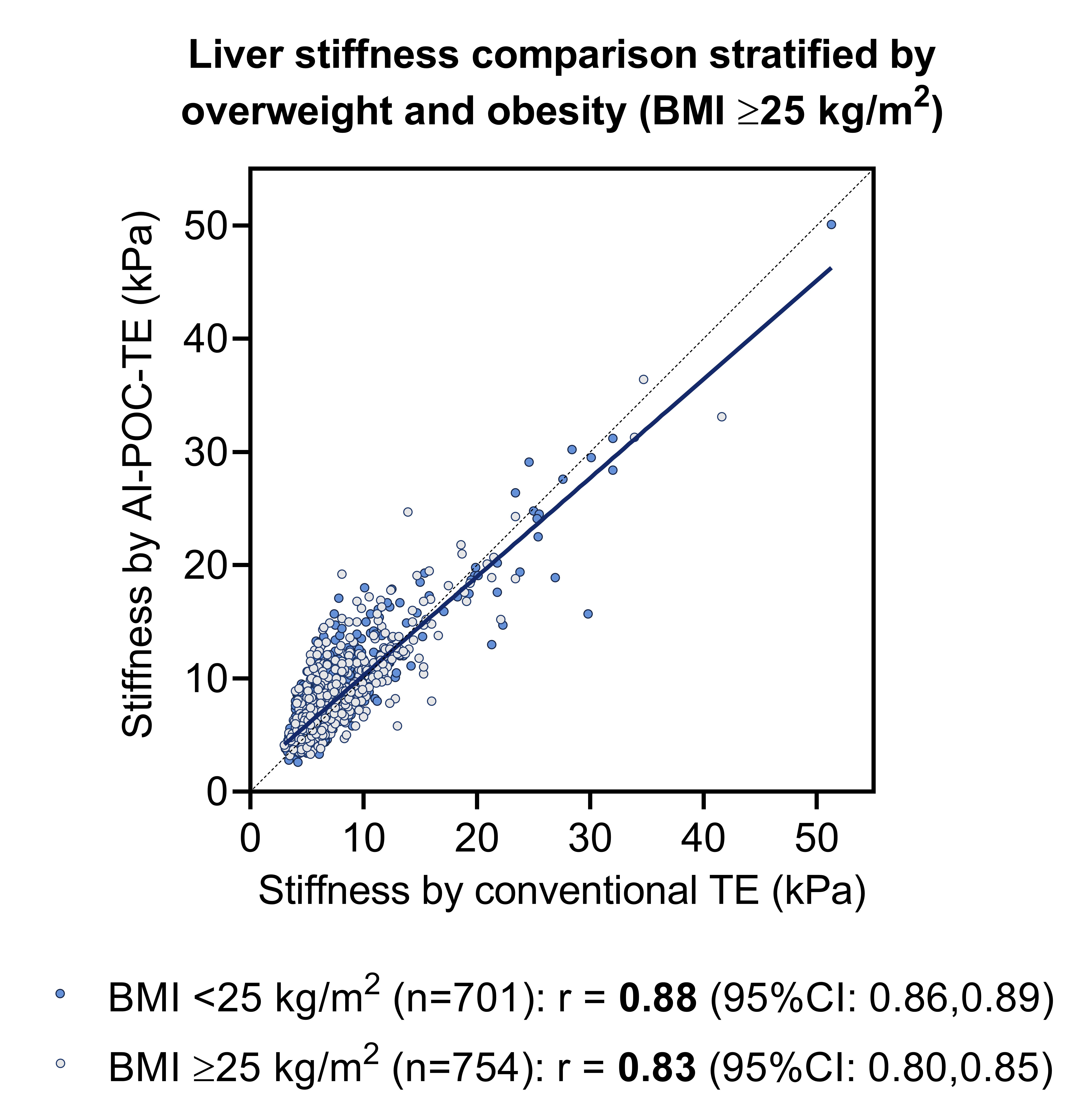


(B)
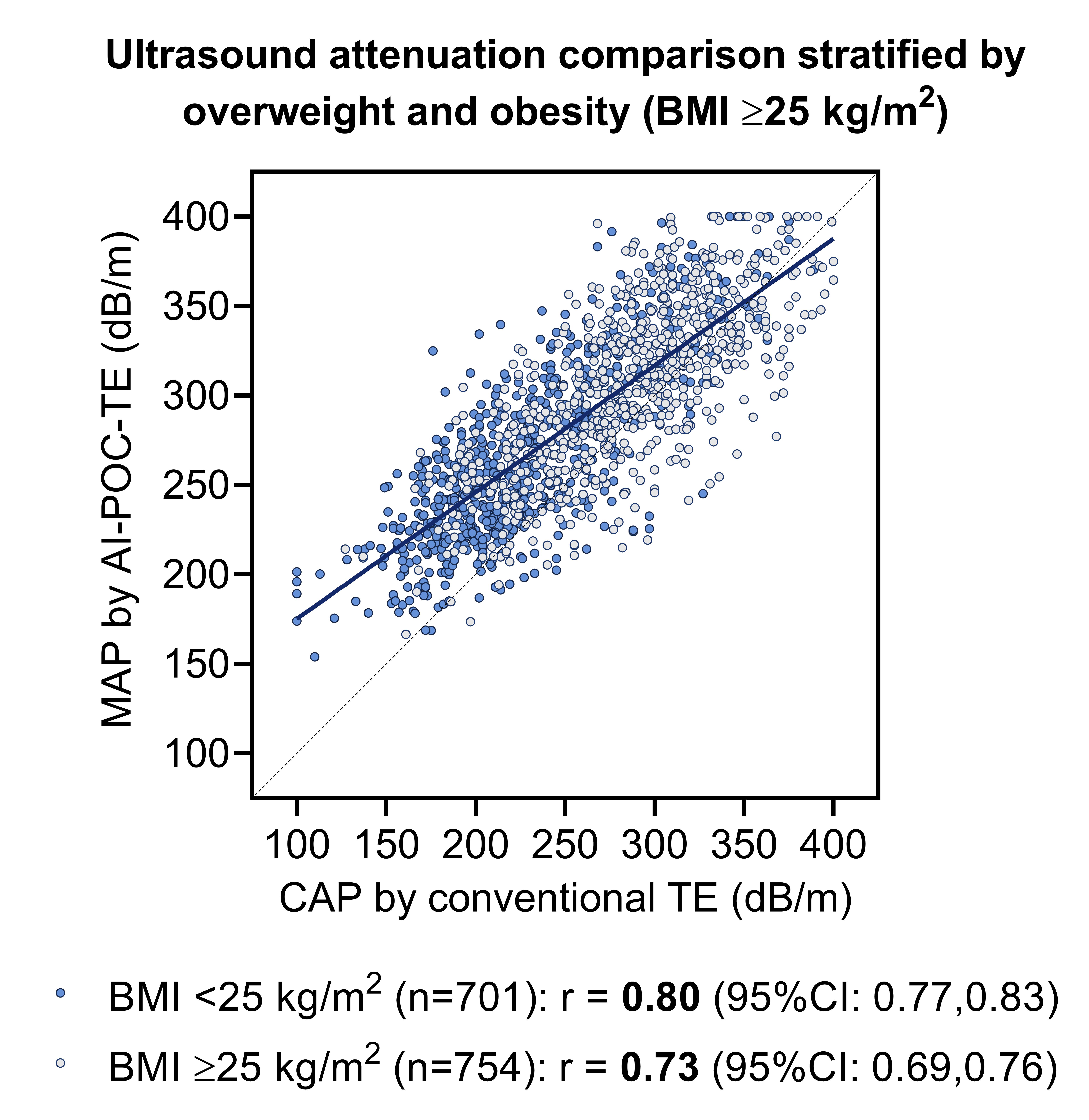


**Supplementary Figure 4. Classification concordance between histology versus Fibroscan**^®^ **and AI-POC-TE in the biopsy cohort, using the cohort–derived optimal cut-offs. (A) Confusion matrix comparing fibrosis stages classified by AI-POC-TE LSM with histologic fibrosis stages (n=138); (B) Confusion matrix comparing fibrosis stages classified by Fibroscan**^®^ **LSM with histologic fibrosis stages (n=138); (C) Confusion matrix comparing steatosis grades classified by AI-POC-TE MAP with histologic steatosis grades (n=136); (D) Confusion matrix comparing steatosis grades classified by Fibroscan**^®^ **CAP with histologic steatosis grades (n=136).**

Staging and grading assignments were based on the optimal cut-offs derived specifically from this biopsy cohort. Diagonal cells represent agreement with the histologic gold standard, and data are presented as number of patients. Overall agreement was calculated as the proportion of patients classified into the same stage as histology. Agreement within ±1 stage or grade was also calculated. Quadratic weighted kappa (κ) was used to evaluate ordinal inter-technique agreement.

(A)
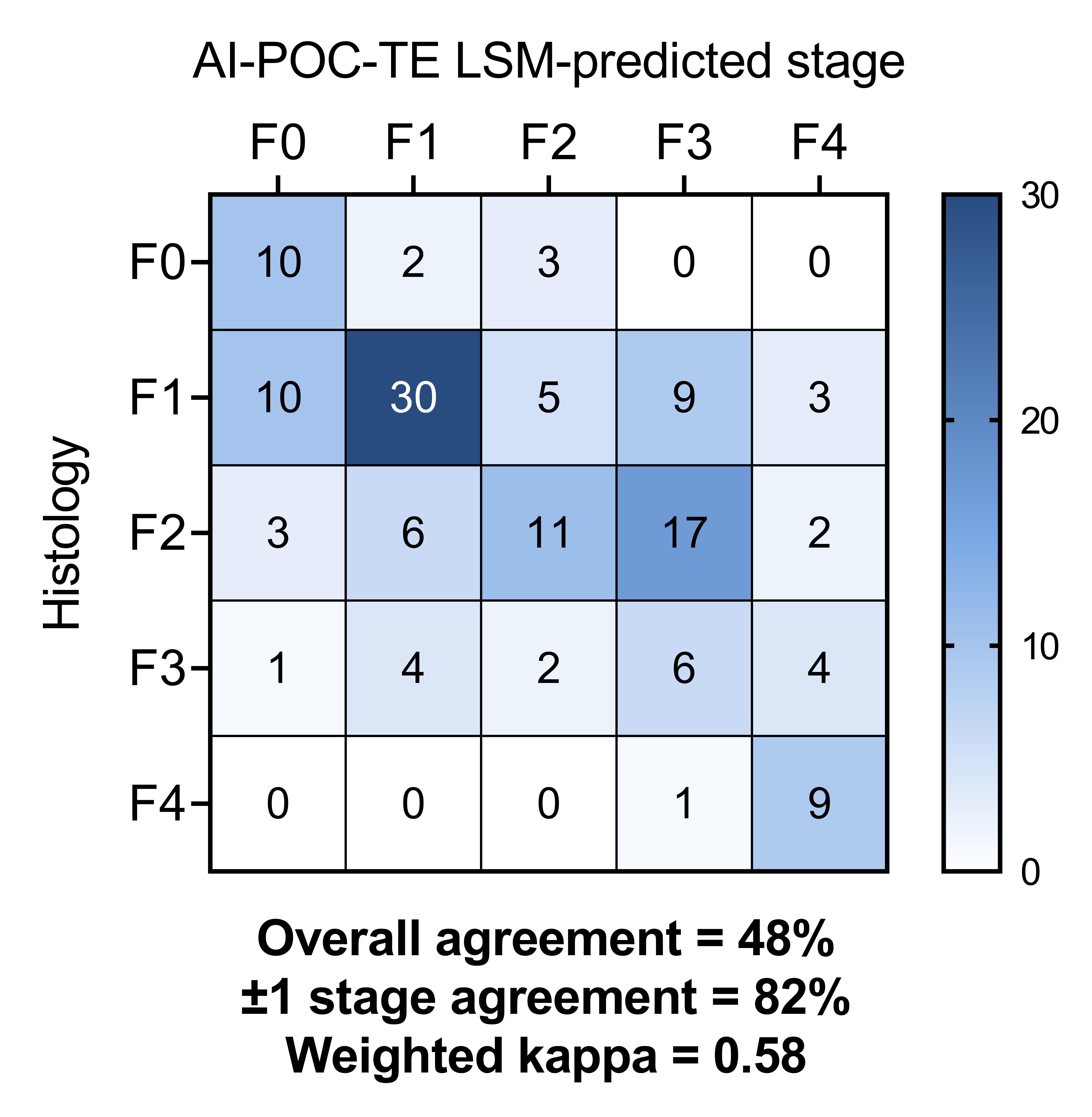
(B)
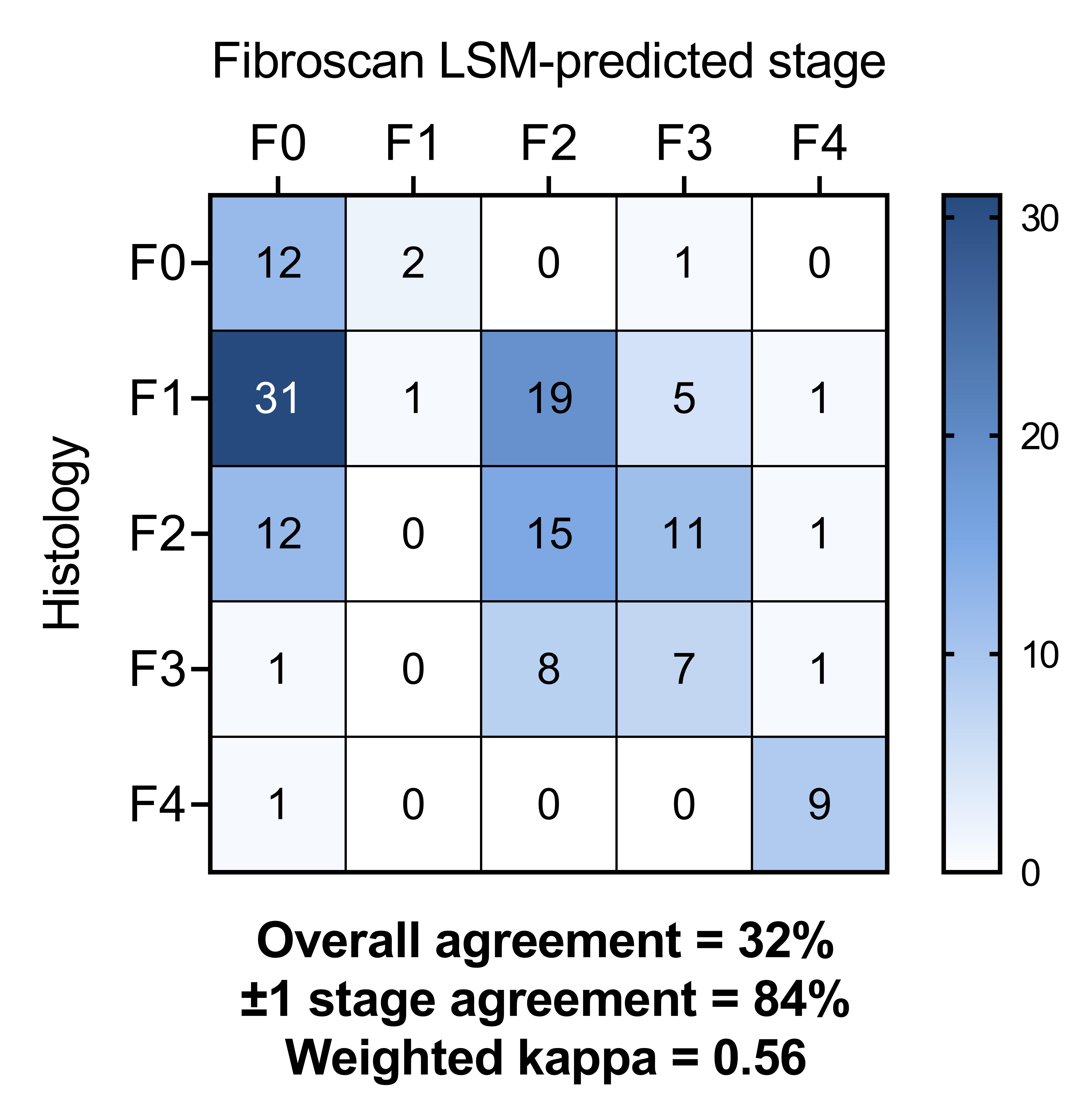


(C)
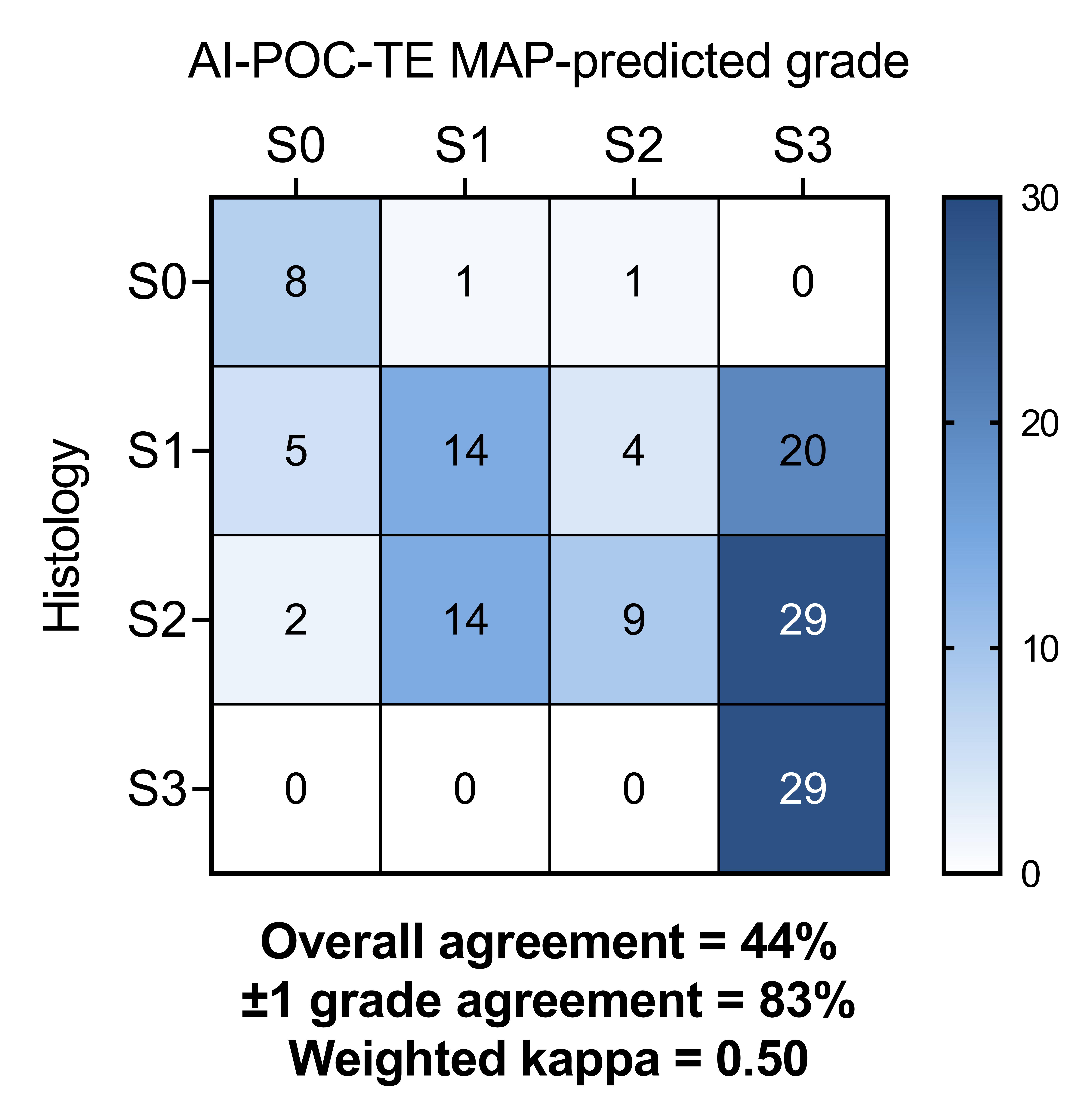
(D)
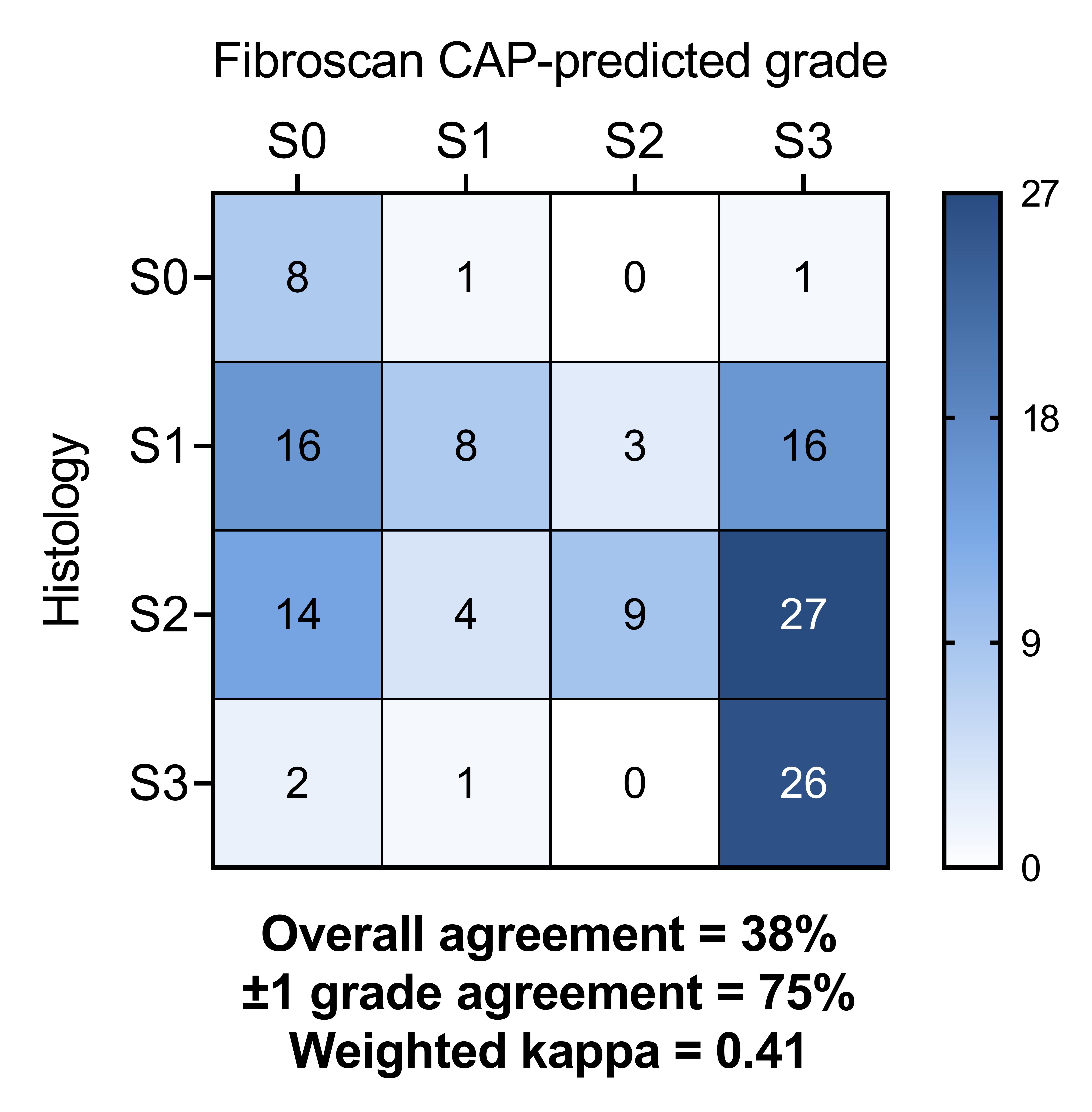


**Supplementary Figure 5. Classification concordance between AI-POC-TE and Fibroscan**^®^ **in the paired TE cohort, using the biopsy cohort–derived optimal cut-offs. (A) Confusion matrix comparing fibrosis stages assigned by AI-POC-TE versus Fibroscan**^®^ **LSM (n=1455); (B) Confusion matrix comparing steatosis grades assigned by AI-POC-TE MAP versus Fibroscan**^®^ **CAP (n=1455).**

Classification for each technique was performed using the optimal cut-offs derived specifically from the biopsy cohort in this study. Crosstabulation is visualized using a heatmap, with darker blue cells indicating higher frequencies. Diagonal cells denote concordance between the two techniques. Overall agreement was calculated as the proportion of patients with identical grading between techniques. Adjacent-stage agreement rate was also reported. Weighted kappa coefficients (quadratic weights) were calculated to assess ordinal agreement.

(A)
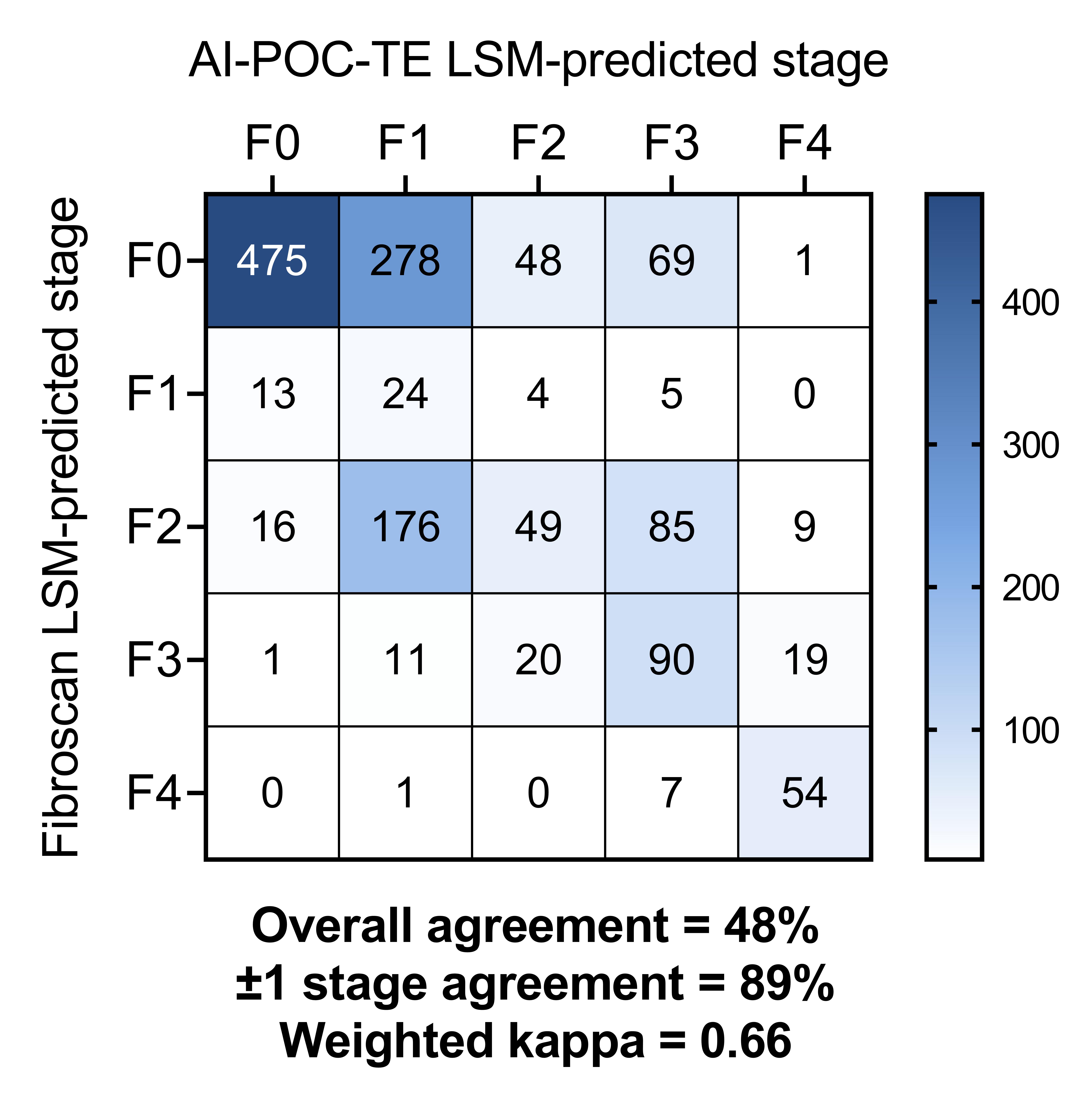
(B)
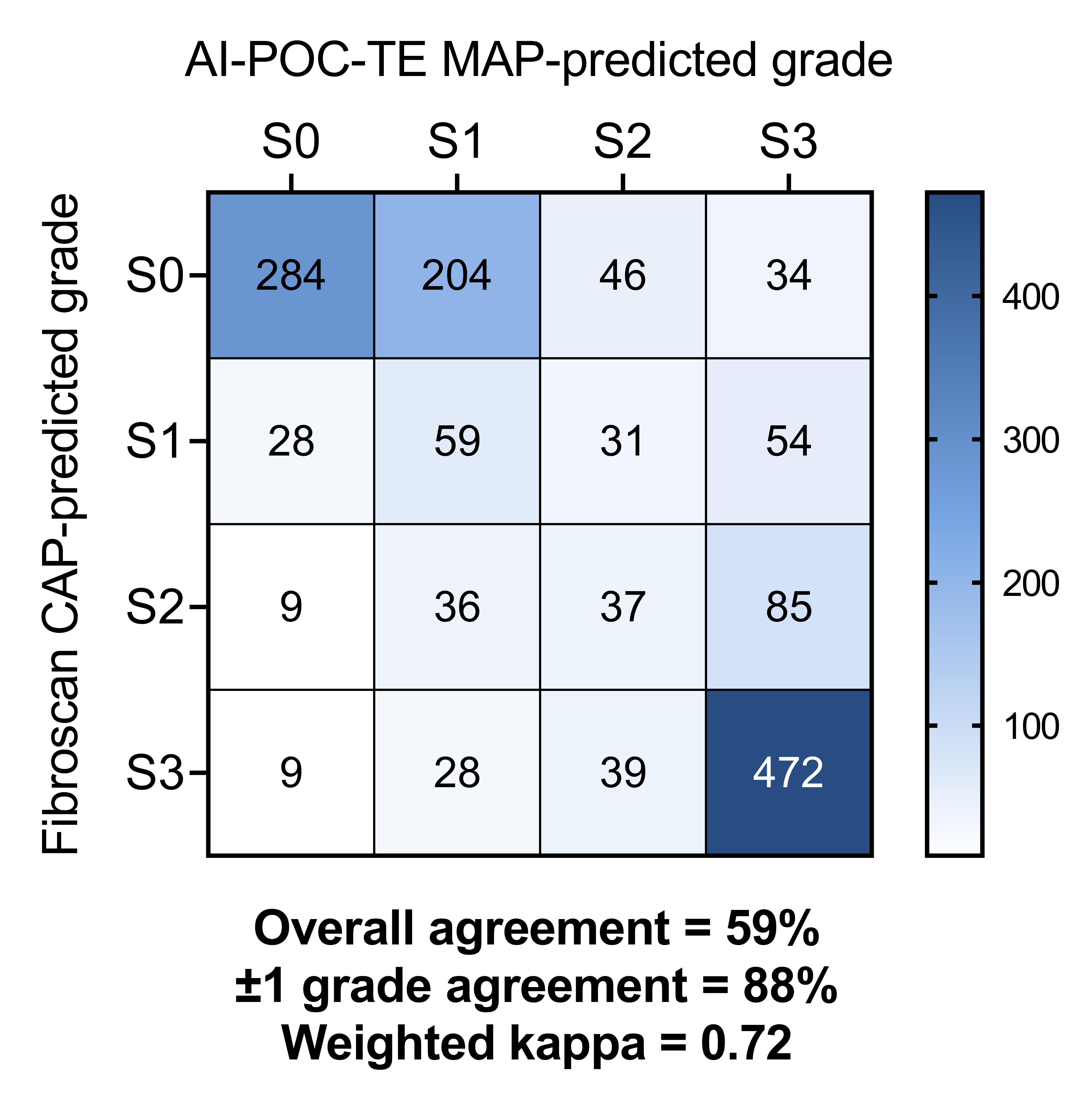


**Supplementary Figure 6. Classification concordance between AI-POC-TE and Fibroscan**^®^ **in the paired TE cohort, using** **the externally established cut-offs reported in the literature. (A) Confusion matrix comparing fibrosis stages assigned by AI-POC-TE versus Fibroscan**^®^ **LSM (n=1455); (B) Confusion matrix comparing steatosis grades assigned by AI-POC-TE MAP versus Fibroscan**^®^ **CAP (n=1455).**

In contrast to the previous analysis (i.e., Supplementary Figure 5, where stage classification was based on study-derived optimal cut-offs), the present analysis applied the cut-offs established in external literature^1,2^ to both modalities in order to evaluate concordance under the same clinically adopted reference criteria. Specifically, fibrosis staging was determined using the common cut-offs for fibrosis severity reported by a meta-analysis study of Tsochatzis et al^2^. Steatosis grading was determined using the CAP thresholds derived from the individual patient data meta-analysis by Karlas et al^1^.

(A)
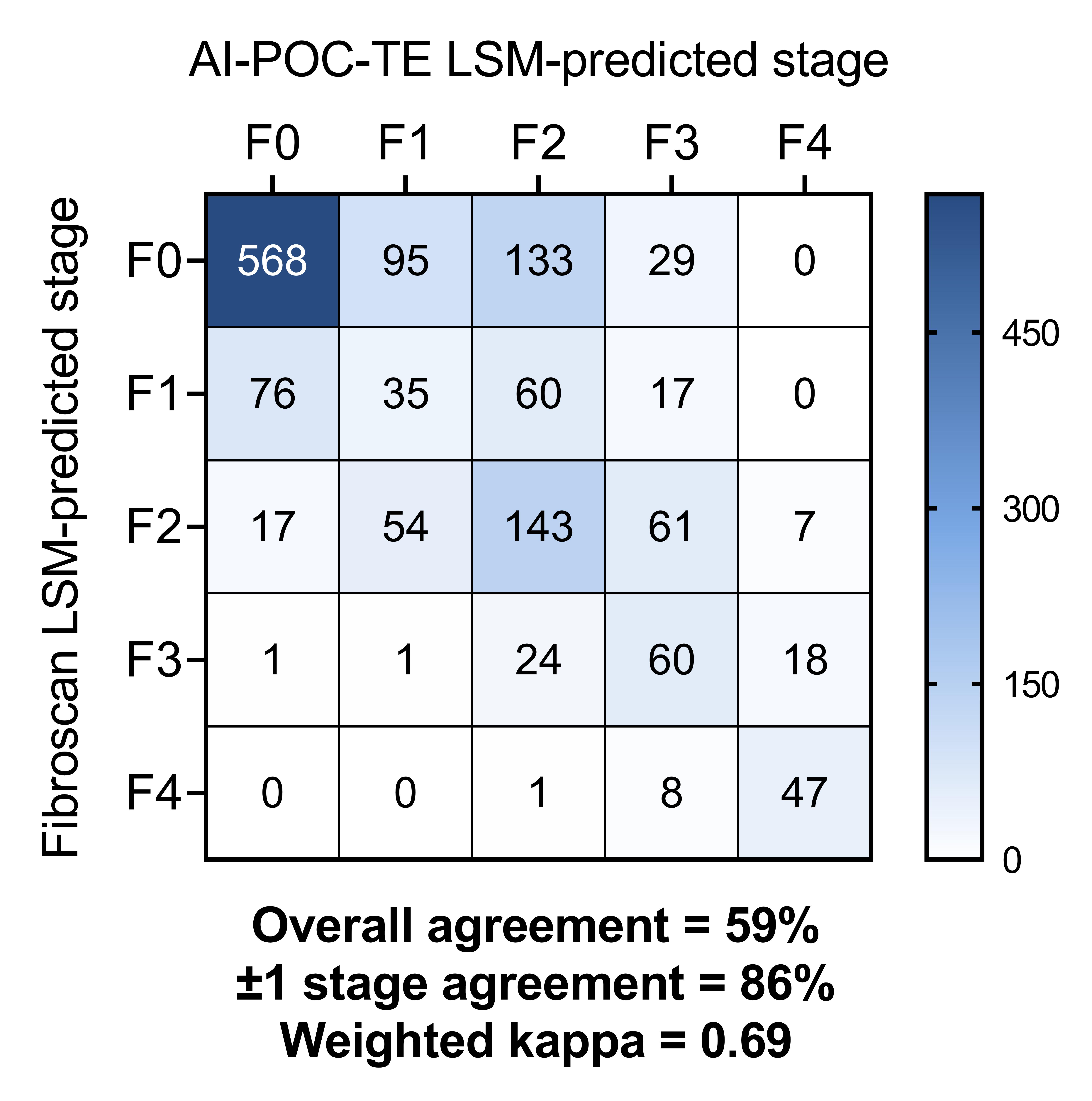
(B)
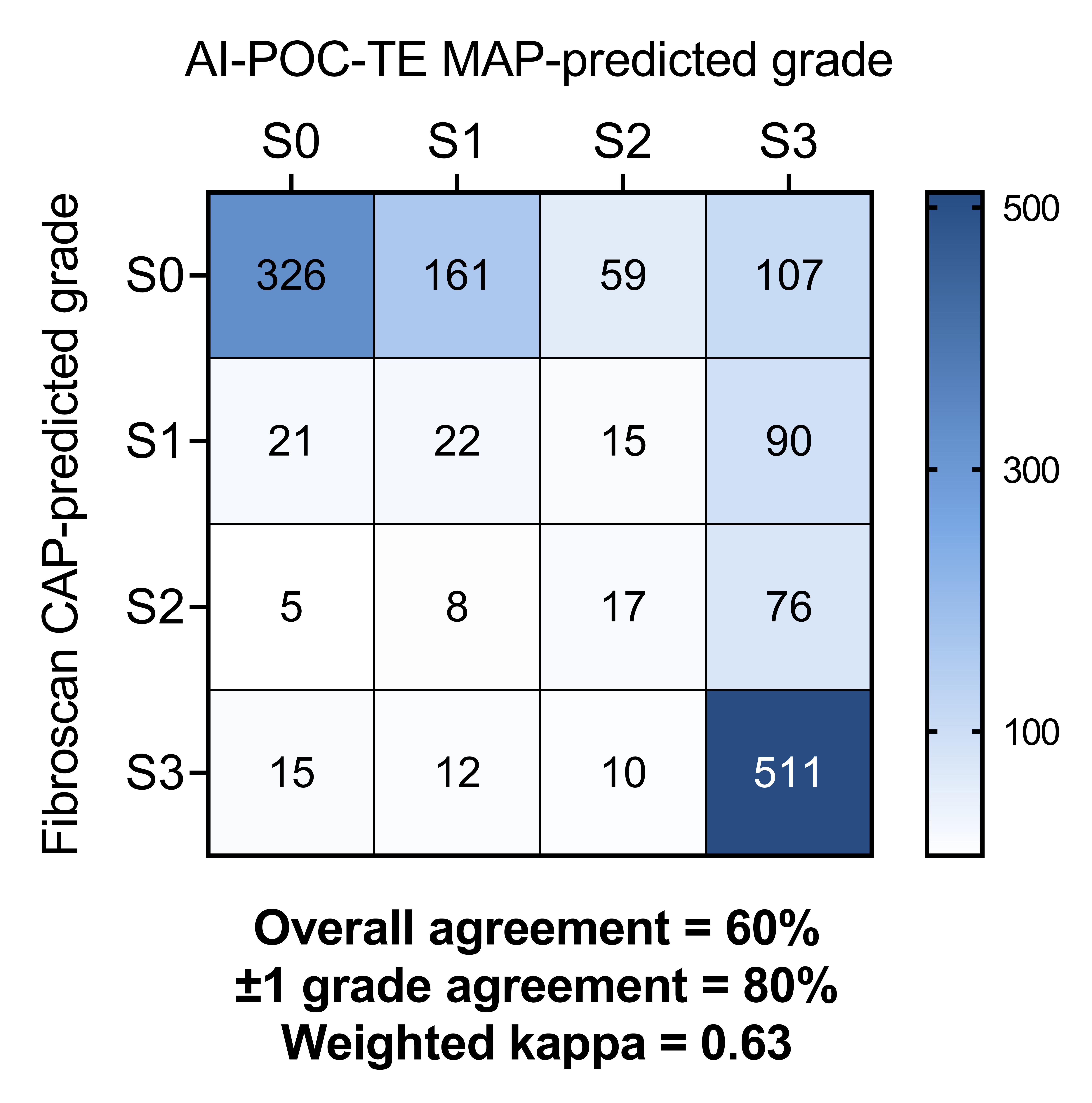


### ***Supplementary Tables***

**Supplementary Table 1. Diagnostic test characteristics of conventional TE (Fibroscan^®^) for hepatic fibrosis and steatosis.**

|  | **Prevalence** | **AUROC**  **(95% CI)** | **Cut-off selection method** | **Cut-off, kPa or dB/m** | **Sensitivity** | **Specificity** |
| --- | --- | --- | --- | --- | --- | --- |
| LSM by Fibroscan^®^ | n = 138 |  |  |  |  |  |
| ≥F1 | 89% | 0.78 (0.70–0.85) | Youden’s index | 6.6 kPa | 0.63 | 0.93 |
| ≥F2 | 48% | 0.76 (0.68–0.83) | Youden’s index | 6.8 kPa | 0.79 | 0.65 |
| ≥F3 | 20% | 0.79 (0.72–0.86) | Youden’s index | 9.5 kPa | 0.63 | 0.85 |
| F4 | 7% | 0.91 (0.85–0.95) | Youden’s index | 14.3 kPa | 0.90 | 0.98 |
|  |  |  |  |  |  |  |
| CAP by Fibroscan^®^ | n = 136 |  |  |  |  |  |
| ≥S1 | 93% | 0.86 (0.79–0.91) | Youden’s index | 237 dB/m | 0.75 | 0.90 |
| ≥S2 | 61% | 0.72 (0.64–0.80) | Youden’s index | 261 dB/m | 0.75 | 0.64 |
| S3 | 21% | 0.75 (0.67–0.82) | Youden’s index | 280 dB/m | 0.90 | 0.60 |

AUROC = area under the receiver operating characteristics curve; LSM = liver stiffness measurement; CAP = controlled attenuation parameter; NPV = negative predictive value; PPV = positive predictive value.

**Supplementary Table 2. Comparative diagnostic accuracy of AI-POC-TE versus conventional TE (Fibroscan^®^) according to liver disease aetiology.**

|  | **Aetiology category** | **AUROC (95% CI)** | **Optimal cut-off** | **Sensitivity** | **Specificity** | ***p*-value* (vs. Fibroscan^®^)** |
| --- | --- | --- | --- | --- | --- | --- |
| **AI-POC-TE:**  USG-LSM for ≥F1 | CHB (n=19) | 0.81 (0.57–0.95) | 5.8 kPa | 0.65 | 1.00 | 0.905 |
|  | MASLD (n=110) | 0.83 (0.74–0.89) | 5.7 kPa | 0.94 | 0.67 | 0.546 |
|  | Whole population (n=138) | 0.83 (0.75–0.89) | 5.8 kPa | 0.89 | 0.67 | 0.481 |
| **Fibroscan^®^:**  LSM for ≥F1 | CHB (n=19) | 0.78 (0.53–0.93) | 5 kPa | 0.65 | 1.00 | - |
|  | MASLD (n=110) | 0.78 (0.69–0.85) | 6.6 kPa | 0.65 | 0.92 | - |
|  | Whole population (n=138) | 0.78 (0.70–0.85) | 6.6 kPa | 0.63 | 0.93 | - |
|  |  |  |  |  |  |  |
| **AI-POC-TE:**  USG-LSM for ≥F2 | CHB (n=19) | 0.64 (0.39–0.85) | 8.1 kPa | 0.63 | 0.82 | 0.738 |
|  | MASLD (n=110) | 0.81 (0.72–0.88) | 7.6 kPa | 0.90 | 0.61 | 0.184 |
|  | Whole population (n=138) | 0.79 (0.71–0.86) | 8.2 kPa | 0.79 | 0.72 | 0.316 |
| **Fibroscan^®^:**  LSM for ≥F2 | CHB (n=19) | 0.61 (0.37–0.82) | 7.0 kPa | 0.50 | 0.91 | - |
|  | MASLD (n=110) | 0.75 (0.66–0.83) | 8.6 kPa | 0.57 | 0.85 | - |
|  | Whole population (n=138) | 0.76 (0.68–0.83) | 6.8 kPa | 0.79 | 0.65 | - |
|  |  |  |  |  |  |  |
| **AI-POC-TE:**  USG-LSM for ≥F3 | CHB (n=19) | 0.79 (0.54–0.94) | 8.1 kPa | 0.80 | 0.79 | 0.441 |
|  | MASLD (n=110) | 0.81 (0.72–0.88) | 9.1 kPa | 0.78 | 0.72 | 0.795 |
|  | Whole population (n=138) | 0.79 (0.72–0.86) | 9.1 kPa | 0.74 | 0.72 | 0.973 |
| **Fibroscan^®^:**  LSM for ≥F3 | CHB (n=19) | 0.83 (0.59–0.96) | 7.0 kPa | 0.80 | 0.93 | - |
|  | MASLD (n=110) | 0.79 (0.70–0.86) | 9.5 kPa | 0.67 | 0.85 | - |
|  | Whole population (n=138) | 0.79 (0.72–0.86) | 9.5 kPa | 0.63 | 0.85 | - |
|  |  |  |  |  |  |  |
| **AI-POC-TE:**  USG-LSM for F4 | CHB (n=19) | 1.00 (0.82–1.00) | 18.7 kPa | 1.00 | 1.00 | 1.000 |
|  | MASLD (n=110) | 0.96 (0.90–0.99) | 11.0 kPa | 1.00 | 0.81 | 0.374 |
|  | Whole population (n=138) | 0.97 (0.93–0.99) | 14.4 kPa | 0.90 | 0.93 | 0.401 |
| **Fibroscan^®^:**  LSM for F4 | CHB (n=19) | 1.00 (0.82–1.00) | 18.9 kPa | 1.00 | 1.00 | - |
|  | MASLD (n=110) | 0.84 (0.76–0.91) | 14.3 kPa | 0.83 | 0.99 | - |
|  | Whole population (n=138) | 0.91 (0.85–0.95) | 14.3 kPa | 0.90 | 0.98 | - |
|  |  |  |  |  |  |  |
| **AI-POC-TE:**  MAP for ≥S1 | CHB (n=18) | 0.90 (0.66–0.99) | 244 dB/m | 0.82 | 0.86 | 0.127 |
|  | MASLD (n=110) | - | - | - | - | - |
|  | Whole population (n=136) | 0.92 (0.86–0.96) | 244 dB/m | 0.94 | 0.80 | 0.250 |
| **Fibroscan^®^:**  CAP for ≥S1 | CHB (n=18) | 0.85 (0.61–0.97) | 204 dB/m | 0.73 | 0.86 | - |
|  | MASLD (n=110) | - | - | - | - | - |
|  | Whole population (n=136) | 0.86 (0.79–0.91) | 237 dB/m | 0.75 | 0.90 | - |
|  |  |  |  |  |  |  |
| **AI-POC-TE:**  MAP for ≥S2 | CHB (n=18) | 0.76 (0.51–0.93) | 244 dB/m | 1.00 | 0.67 | 0.352 |
|  | MASLD (n=110) | 0.64 (0.54–0.73) | 300 dB/m | 0.67 | 0.62 | 0.636 |
|  | Whole population (n=136) | 0.70 (0.61–0.77) | 278 dB/m | 0.82 | 0.53 | 0.502 |
| **Fibroscan^®^:**  CAP for ≥S2 | CHB (n=18) | 0.83 (0.59–0.96) | 198 dB/m | 1.00 | 0.58 | - |
|  | MASLD (n=110) | 0.66 (0.57–0.75) | 261 dB/m | 0.75 | 0.59 | - |
|  | Whole population (n=136) | 0.72 (0.64–0.80) | 261 dB/m | 0.75 | 0.64 | - |
|  |  |  |  |  |  |  |
| **AI-POC-TE:**  MAP for S3 | CHB (n=18) | 0.75 (0.49–0.92) | 293 dB/m | 1.00 | 0.75 | 0.143 |
|  | MASLD (n=110) | 0.74 (0.65–0.82) | 294 dB/m | 1.00 | 0.49 | 0.914 |
|  | Whole population (n=136) | 0.76 (0.68–0.83) | 294 dB/m | 1.00 | 0.54 | 0.962 |
| **Fibroscan^®^:**  CAP for S3 | CHB (n=18) | 0.88 (0.64–0.98) | 280 dB/m | 1.00 | 0.88 | - |
|  | MASLD (n=110) | 0.73 (0.64–0.81) | 285 dB/m | 0.89 | 0.58 | - |
|  | Whole population (n=136) | 0.75 (0.67–0.82) | 280 dB/m | 0.90 | 0.60 | - |

The optimal cut-off values were determined using the Youden’s index. Because none of the 110 patients with MASLD had a histological diagnosis of steatosis grade 0 (i.e., S0), ROC analyses of CAP and MAP for ≥S1 were not feasible in the MASLD subgroup.

AUROC = area under the receiver operating characteristics curve; AI-POC-TE = artificial intelligence-enabled point-of-care transient elastography; CHB = chronic hepatitis B; USG-LSM = ultrasonography-guided liver stiffness measurement; MAP = multi-domain attenuation parameter; MASLD = metabolic dysfunction-associated steatotic liver disease.

*AUROC comparison of AI-POC-TE versus Fibroscan^®^ within the same aetiology category using DeLong’s test.

**Supplementary Table 3. Comparative diagnostic accuracy of AI-POC-TE as function of reliability criteria.**

|  | **Reliability criteria** | **AUROC (95% CI)** |
| --- | --- | --- |
| USG-LSM for ≥F2 | IQR/median ratio ≤30% (n=89) | 0.78 (0.68–0.88) |
|  | IQR/median ratio >30% (n=49) | 0.80 (0.67–0.93) |
|  | Whole population (n=138) | 0.79 (0.71–0.86) |
|  |  |  |
| USG-LSM for ≥F3 | IQR/median ratio ≤30% (n=89) | 0.81 (0.70–0.92) |
|  | IQR/median ratio >30% (n=49) | 0.75 (0.57–0.94) |
|  | Whole population (n=138) | 0.79 (0.72–0.86) |
|  |  |  |
| MAP for ≥S2 | IQR ≤40 dB/m (n=114) | 0.69 (0.58–0.80) |
|  | IQR >40 dB/m (n=22) | 0.67 (0.42–0.91) |
|  | Whole population (n=136) | 0.70 (0.61–0.77) |
|  |  |  |
| MAP for S3 | IQR ≤40 dB/m (n=114) | 0.80 (0.73–0.88) |
|  | IQR >40 dB/m (n=22) | 0.53 (0.00–1.00) |
|  | Whole population (n=136) | 0.76 (0.68–0.83) |

AUROC = area under the receiver operating characteristics curve; AI-POC-TE = artificial intelligence-enabled point-of-care transient elastography; USG-LSM = ultrasonography-guided liver stiffness measurement; MAP = multi-domain attenuation parameter; IQR = interquartile range.

**Supplementary Table 4. Comparative diagnostic accuracy of AI-POC-TE and conventional TE (Fibroscan^®^) as function of BMI category.**

|  | **BMI criteria** | **AUROC (95% CI)** |
| --- | --- | --- |
| **AI-POC-TE:** USG-LSM for ≥F2 | BMI <25 kg/m^2^ (n=41) | 0.80 (0.66–0.94) |
|  | BMI ≥25 kg/m^2^ (n=97) | 0.79 (0.69–0.88) |
|  | Whole population (n=138) | 0.79 (0.71–0.86) |
|  |  |  |
| **Fibroscan^®^:** LSM for ≥F2 | BMI <25 kg/m^2^ (n=41) | 0.76 (0.60–0.91) |
|  | BMI ≥25 kg/m^2^ (n=97) | 0.76 (0.66–0.85) |
|  | Whole population (n=138) | 0.76 (0.68–0.83) |
|  |  |  |
| **AI-POC-TE:** USG-LSM for ≥F3 | BMI <25 kg/m^2^ (n=41) | 0.85 (0.70–0.99) |
|  | BMI ≥25 kg/m^2^ (n=97) | 0.78 (0.66–0.90) |
|  | Whole population (n=138) | 0.79 (0.72–0.86) |
|  |  |  |
| **Fibroscan^®^:** LSM for ≥F3 | BMI <25 kg/m^2^ (n=41) | 0.87 (0.72–1.00) |
|  | BMI ≥25 kg/m^2^ (n=97) | 0.76 (0.62–0.89) |
|  | Whole population (n=138) | 0.79 (0.72–0.86) |
|  |  |  |
| **AI-POC-TE:** MAP for ≥S1 | BMI <25 kg/m^2^ (n=40) | 0.89 (0.77–1.00) |
|  | BMI ≥25 kg/m^2^ (n=96) | 0.89 (0.75–1.00) |
|  | Whole population (n=136) | 0.92 (0.86–0.96) |
|  |  |  |
| **Fibroscan^®^:** CAP for ≥S1 | BMI <25 kg/m^2^ (n=40) | 0.78 (0.60–0.95) |
|  | BMI ≥25 kg/m^2^ (n=96) | 0.89 (0.76–1.00) |
|  | Whole population (n=136) | 0.86 (0.79–0.91) |
|  |  |  |
| **AI-POC-TE:** MAP for S3 | BMI <25 kg/m^2^ (n=40) | 0.92 (0.83–1.00) |
|  | BMI ≥25 kg/m^2^ (n=96) | 0.69 (0.58–0.79) |
|  | Whole population (n=136) | 0.76 (0.68–0.83) |
|  |  |  |
| **Fibroscan^®^:** CAP for S3 | BMI <25 kg/m^2^ (n=40) | 0.89 (0.78–0.99) |
|  | BMI ≥25 kg/m^2^ (n=96) | 0.70 (0.58–0.82) |
|  | Whole population (n=136) | 0.75 (0.67–0.82) |

AUROC = area under the receiver operating characteristics curve; AI-POC-TE = artificial intelligence-enabled point-of-care transient elastography; BMI = body mass index; USG-LSM = ultrasonography-guided liver stiffness measurement; MAP = multi-domain attenuation parameter.

# References

1. Karlas T, Petroff D, Sasso M, et al. Individual patient data meta-analysis of controlled attenuation parameter (CAP) technology for assessing steatosis. *Journal of hepatology*. 2017;66(5):1022-1030. doi:10.1016/j.jhep.2016.12.022

2. Tsochatzis EA, Gurusamy KS, Ntaoula S, Cholongitas E, Davidson BR, Burroughs AK. Elastography for the diagnosis of severity of fibrosis in chronic liver disease: A meta-analysis of diagnostic accuracy. *Journal of hepatology*. 2011;54(4):650-659. doi:10.1016/j.jhep.2010.07.033
